# Factors influencing the development, recruitment, integration, retention and career development of advanced practice providers in hospital health care teams: a scoping review

**DOI:** 10.1186/s12916-024-03509-6

**Published:** 2024-07-08

**Authors:** Yingxi Zhao, Wesley Quadros, Shobhana Nagraj, Geoff Wong, Mike English, Attakrit Leckcivilize

**Affiliations:** 1 Nuffield Department of Medicine Centre for Global Health Research, University of Oxford, S Parks Rd, Oxford, OX1 3SY UK; 2 East & North Hertfordshire NHS Trust, Stevenage, UK; 3 Department of Public Health & Primary Care, University of Cambridge, Cambridge, UK; 4 Nuffield Department of Primary Care Health Sciences, University of Oxford, Oxford, UK; 5 KEMRI-Wellcome Trust Research Programme, Nairobi, Kenya

**Keywords:** Secondary care, Extended roles, Additional roles, Physician assistant/associate, Nurse practitioner, Employee management, Teamwork

## Abstract

**Background:**

Advanced practice providers (APPs), including physician assistants/associates (PAs), nurse practitioners (NPs) and other non-physician roles, have been developed largely to meet changing healthcare demand and increasing workforce shortages. First introduced in primary care in the US, APPs are prevalent in secondary care across different specialty areas in different countries around the world. In this scoping review, we aimed to summarise the factors influencing the development, recruitment, integration, retention and career development of APP roles in hospital health care teams.

**Methods:**

We conducted a scoping review and searched Ovid MEDLINE, Ovid Embase, Ovid Global Health, Ovid PsycINFO and EBSCOhost CINAHL to obtain relevant articles published between Jan 2000 and Apr 2023 that focused on workforce management of APP roles in secondary care. Articles were screened by two reviewers independently. Data from included articles were charted and coded iteratively to summarise factors influencing APP development, recruitment, integration, retention and career development across different health system structural levels (macro-, meso- and micro-level).

**Results:**

We identified and analysed 273 articles that originated mostly from high-income countries, e.g. the US (*n* = 115) and the UK (*n* = 52), and primarily focused on NP (*n* = 183) and PA (*n* = 41). At the macro-level, broader workforce supply, national/regional workforce policies such as work-hour restrictions on physicians, APP scope of practice regulations, and views of external collaborators, stakeholders and public representation of APPs influenced organisations’ decisions on developing and managing APP roles. At the meso-level, organisational and departmental characteristics, organisational planning, strategy and policy, availability of resources, local experiences and evidence as well as views and perceptions of local organisational leaders, champions and other departments influenced all stages of APP role management. Lastly at the micro-level, individual APPs’ backgrounds and characteristics, clinical team members’ perceptions, understanding and relationship with APP roles, and patient perceptions and preferences also influenced how APPs are developed, integrated and retained.

**Conclusions:**

We summarised a wide range of factors influencing APP role development and management in secondary care teams. We highlighted the importance for organisations to develop context-specific workforce solutions and strategies with long-term investment, significant resource input and transparent processes to tackle evolving healthcare challenges.

**Supplementary Information:**

The online version contains supplementary material available at 10.1186/s12916-024-03509-6.

## Background

Increasing demand, growing complexity of healthcare and workforce shortages have led hospitals and healthcare organisations to consider innovative service models and new clinical roles [1, 2]. Advanced practice providers (APPs), including physician assistants/associates (PAs), nurse practitioners (NPs) and other non-physician roles, is a term that has been used for coding and billing purposes by the US Medicare and Medicaid [3]; however, we acknowledge that this term might not be accepted or preferred by all PA and NP professions but use it for convenience here. APPs have a long history, dating back as early as the nineteenth century in low- and middle-income countries [4]. They later emerged in the US in the 1960s and subsequently in many countries of various stages of development [5, 6]. Now PAs exist in over 62 countries under different practice titles [7], and an estimated 40 countries have well-established NP roles [8]. Both PA and NP roles were first introduced in primary care in the US to address the shortage of primary care physicians, but their roles have evolved and they are now more prevalent in secondary care and across different specialty areas [9, 10]. Despite variable job role titles, APPs often have considerable overlap in their scope of practice, often including providing clinical leadership and enabling collaborations in multi-professional teams [11, 12].

Previous systematic reviews have provided evidence on the role and contribution of APPs in hospital health care teams, including reduced waiting and processing time, improved patient continuity and satisfaction, similar clinical safety and patient outcomes, and possibly contained cost and enhanced educational experiences of other trainees [13–19]. There has been less focus on understanding why and how health systems and hospitals develop APP roles as well as how to best integrate and retain them. This is especially important as reconfiguring the workforce and introducing new roles will often lead to professional jurisdiction and boundary tensions, impacting early role development and integration [20]. Additionally, less well-defined career progression pathways beyond entry-level especially for roles like PA [21] could lead to poor retention, worsening workforce shortages and avoidable turnover costs.

In this scoping review, we aim to summarise the factors associated with the development, recruitment, integration, retention and career development of APP roles in secondary care teams. This review can inform regulators, hospital managers and policy-makers on factors to consider while using APPs to address hospital workforce challenges. This is especially important for countries like the UK where the development of PA roles is relatively new and hospitals face significant challenges integrating PA into the workforce with confusion over and contestation around roles.

## Methods

We followed the five steps of Arksey and O’Malley method [22] for scoping reviews to capture a broad range of research evidence from the academic literature. Our research question is ‘what influenced the development, recruitment, integration, retention and career development of APPs into secondary care teams?’

### Search strategy and screening

In consultation with an experienced librarian, we conducted a systematic search using Ovid MEDLINE, Ovid Embase, Ovid Global Health, Ovid PsycINFO and EBSCOhost CINAHL to obtain relevant articles. We included all kinds of study designs as well as case reports published between Jan 2000 and Apr 2023. We combined keyword terms and phrases related to NP, PA, workforce, secondary and hospital care (see Additional file 1 for an example search strategy). We are interested in roles like PAs, NPs and equivalent professions in other countries (clinical officers, assistant medical officers, surgical care practitioners, anaesthesia assistants, etc.) where the primary aim is to share work with doctors formally. We included articles if they examined any aspect of role development, recruitment, integration, retention and career development (see Table 1 for definitions for each focus) of APPs in secondary care and above, including hospital-based ambulatory care. We excluded articles that primarily focus on primary care and community health or were not empirical work.

**Table 1:** Definitions used for the scoping review

• Focus—we are interested in the following aspects of workforce management:
o Development refers to when organisations start a completely new role
o Recruitment refers to when organisations identify, attract, interview, select and hire new workers
o Integration refers to enabling workers to understand each other’s roles and contributions, and building support networks around individuals and their community [23]. We include the onboarding and orientation process as part of the integration
o Retention refers to keeping and continue employing existing workers
o Career development refers to enabling individual workers’ professional growth
• Roles—we recognise different APP roles have different titles; in this review, we have grouped the articles into the following categories:
o Physician associates (PA) including physician assistants
o Nurse practitioners (NP) including advanced nurses, nurse specialists and nurse consultants
o Clinical officers commonly seen in low- and middle-income countries
o Others including anaesthesia associates and surgical care practitioners
• Levels—we summarise findings across different health system structural levels:
o Macro-level refers to system-wide issues and those external to the organisation
o Meso-level refers to organisational and departmental issues
o Micro-level refers to individual and interpersonal issues

**Fig. 1:**
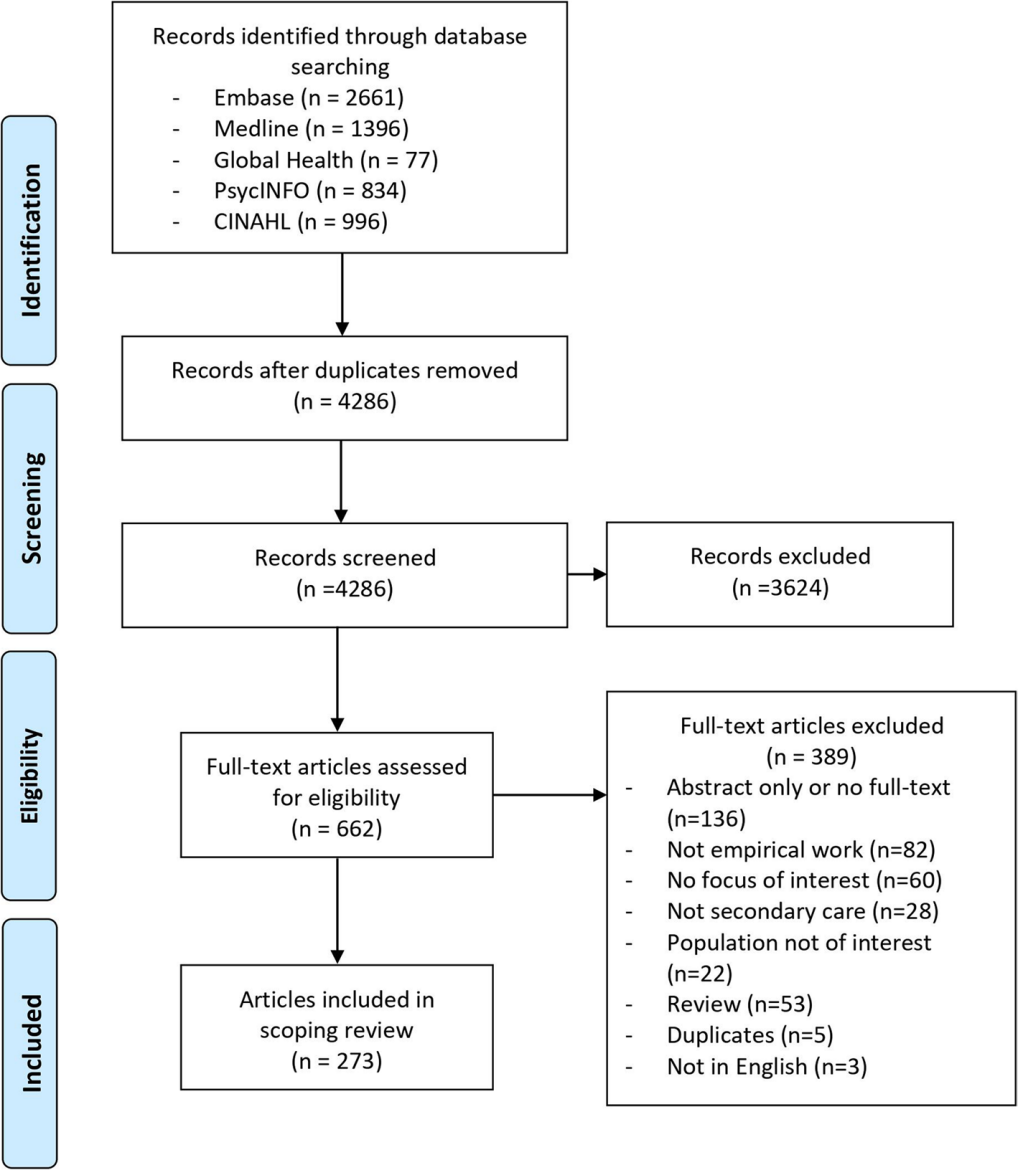
PRISMA diagram

After deduplication, we imported the citations into an online screening tool (Abstrackr) for the initial title and abstract screening [24]. Two reviewers were involved in the title and abstract, and full-text screening. YZ reviewed all the titles and abstracts to assess eligibility for full-text review, and a random subset of 20% was reviewed independently by WQ. Full texts were reviewed by both YZ and WQ independently to determine inclusion. We resolved disagreements on inclusion at the title and abstract stage or at the full-text stage through discussion between the two reviewers. The PRISMA flow diagram is shown in Fig. 1.

### Data charting and collation

YZ first charted data from included articles and entered them into a Microsoft Excel spreadsheet. We extracted data on the year of publication, country of focus, clinical setting (inpatient, outpatient, ambulatory, emergency or mixed settings), healthcare professional type (PA, NP, others or mixed cadres of staff), study design and participant characteristics. Included articles were then coded by YZ using NVivo (version 1.5.1) focusing on factors influencing the development, recruitment, integration, retention and career development of APPs. We used a thematic and result-based convergent mixed-methods approach [25] to understand and triangulate factors at different health system levels (see Table 1). The coding process is largely interpretive, using both first-order (participants’ own words) and second-order constructs (researchers’ interpretation) [26] to develop understanding, especially as many articles did not specifically focus on integration but had relevant data.

### Summarising and reporting findings

We categorised factors into three stages of workforce management (development and recruitment, integration, and retention and career development) and three health system levels (macro, meso and micro). Core thematic areas and subthemes were refined and summarised iteratively within each category. New themes that emerged from the coding analysis but did not fit in any prior themes were listed separately. Preliminary findings were presented to the other authors who were not directly involved in coding to triangulate and increase the trustworthiness of the findings. The PAGER (Patterns, Advances, Gaps, Evidence for practice and Research recommendations) framework was also used to summarise major gaps and recommendations [27].

The review team members are primarily health systems researchers and clinicians working on a project focusing on PAs in NHS hospitals. While none of the members is professionally aligned with any type of APPs, the review findings were also presented to a broad network of PA stakeholders in the UK including PAs, clinicians working with PAs, educators, hospital managers and regulators, to further sense-check the relevance of these findings. The combination of outsider-insider views further validates our understanding of the topic.

## Results

### Article overview

Of 4286 citations identified after deduplication, 273 met inclusion criteria after the full-text review. The characteristics of the included articles are provided in Table 2 and Additional file 2. The majority of articles focused on high-income countries and territories, especially US (*n* = 115) and UK (*n* = 52), whereas only 7 focused on low- and middle-income countries, including Malawi, Zambia, Uganda and Kenya. Sixty-seven percent (*n* = 183) of articles focused on NPs, 15% (*n* = 41) focused on PAs and the rest focused on a mix of different cadres or other cadres like anaesthesia associates. One hundred and two articles discussed the development and recruitment of APPs, 221 discussed integration and only 43 articles analysed the retention and career development of APPs. Major thematic areas that summarised different factors are presented in Table 3 and explained below. The full list of subthemes with supporting data is provided in Additional file 3. Main findings, associated research gaps and future research recommendations are provided in Additional file 4.

**Table 2:** Summary of included articles characteristics

**Characteristics**	**Number (%)**
**Country or territory**	
US	115 (42%)
UK	52 (19%)
Canada	25 (9%)
Australia	21 (8%)
Netherlands	11 (4%)
Ireland	7 (3%)
Taiwan	6 (2%)
Others	36 (13%)
**Year of publication**	
2020–2023	61 (22%)
2010–2019	152 (56%)
2000–2009	60 (22%)
**Study design**	
Quantitative	128 (47%)
Qualitative	117 (43%)
Mixed methods	28 (10%)
**Professional role/cadre of focus**	
Nurse practitioner	183 (67%)
Physician assistant/associate	41 (15%)
Clinical officer	7 (3%)
Mixed cadre	38 (14%)
Others	4 (1%)
**Setting**	
Inpatient	91 (33%)
Outpatient	18 (7%)
Emergency	51 (19%)
Ambulatory	1 (0.3%)
Other mixed setting	112 (41%)
**Focus**	
Development and recruitment	102 (37%)
Integration	221 (81%)
Retention and career development	43 (16%)

**Table 3:** Summary table of factors influencing the development, recruitment, integration, retention and career development of advanced practice providers (APPs)

**Level**	**Development and recruitment**	**Integration**	**Retention and career development**
**Macro (system-wide)**	• APP and other workforce supply • National or regional workforce policy • Views of external stakeholders and collaborators	• National or regional scope of work and service reimbursement policy • APP representation outside of organisations	• Career recognition, structure and pathways
**Meso (organisational and departmental)**	• Characteristics of hospitals and departments • Organisational need and planning • Organisational policy and arrangement • Resources and processes for role development and recruitment • Local experience and evidence • Organisational leaders’ or champions’ perception and understanding • Views of other departments and teams	• Organisational culture • Organisational strategy and planning • Organisational policy and arrangement • Resources to support role activities and integration • Clinical training resource and opportunities • Local experience and evidence • Organisational leaders’ or champions’ perception and understanding • Views of other departments and teams • APP representation in organisations	• Organisational culture • Organisational strategy and planning • Organisational policy and arrangement • Resources and opportunities for continued employment and career development • Organisational leaders’ attitude
**Micro (individual and interpersonal)**	• APP individual interest and intention • Clinical team members’ perception and understanding • Patient perception or preference	• APP individual background and attribute • APP individual skills and expertise • Relationships and negotiations with clinical team members and peers • Autonomy and relationships with supervisors • Patient perception or preference of APP	• APP individual background • APP work experience and beliefs • Relationship with clinical team members and peers

### Macro-level system-wide factors

We found that the *broader workforce supply* influenced organisations’ decisions on whether or not to develop and recruit APPs. Whereas many countries have an increasing supply of APPs, in some cases there was a relative shortage of APPs with specific specialty training and this impeded organisations’ development and recruitment decisions [28–30]. There were also examples of an increasing supply of medical students and residents with hospitals concerned that recruiting APPs would lead to competition for training opportunities [31, 32].

*National or regional workforce policies* played important roles in APP development and recruitment decisions and also impacted whether or not APPs could be fully integrated into clinical teams. While the national or regional government might issue broad policy guidance or have specific funding streams encouraging APP role development [30, 33–36], their relatively limited scope of practice and regulations for prescription [37–39], billing and service reimbursement [39, 40] led to organisations’ hesitancy to develop and recruit APPs, or when they were recruited their integration was more challenging [32, 41–43]. Workforce policies such as work-hour restrictions on medical trainees encouraged organisations to consider developing and recruiting APP roles [44–47]. Lastly, whether national workforce policies and regulators recognise APP role titles especially those with further specialised training [48, 49] and whether there is a clear and well-developed career progression pathway in the system [30, 50, 51] impacted APPs’ retention and career development.

Similarly important for recruitment, development and integration are *views of external collaborators, stakeholders, as well as public representation of APPs*. This included, for example, support or opposition from professional associations of physicians [37], or from other hospital collaborators such as specific charities [52, 53]. How APPs are being represented outside of organisations such as public images and advocacy for APPs also influenced whether they could be successfully integrated into clinical teams [54, 55].

### Meso-level organisational and departmental factors

*Specific hospital characteristics* influenced decisions to develop and recruit APPs, for example academic hospitals affiliated with APP training institutions and rural facilities that have challenges in staffing were reported to be more likely to consider developing and recruiting APPs [39, 56].

Many articles highlighted the importance of *organisational planning and strategy* in APP role development, recruitment, integration, retention and career development. The organisational view of existing workforce shortages [37, 57], service gaps [58, 59] and anticipated roles and values [30, 44, 60–62] of APPs often influenced whether APPs were recruited and their specific category. For example, this included organisations aiming for service expansion requiring less specialised APPs to meet the increasing demand for lower acuity patients [60], those intending that APPs take on more quality improvement roles [30, 63] when hospital ratings were assessed as low by regulators. While APPs were often prioritised for filling rotas, improving patient continuity and holistic care [30, 40, 42, 64, 65], sometimes there could be disagreement between hospital leadership over role priorities that impeded role enactment and integration [66], or APPs might be prioritised for clinical functions only without supporting them as individuals which could lead to dissatisfaction and poor retention [67, 68]. Often, organisational planning and strategy are linked with *organisational leaders’ or champions’ perception and understanding* of APP roles. Poor understanding of what APPs are, concerns over autonomy and working hour arrangements sometimes impeded role development, integration and retention [37, 44, 68–71], whereas key champions and advocates from the organisation and departments played a huge role in facilitating role development, recruitment and integration [66, 72–75]. *Local experiences and evidence* of APPs were relevant to the development and integration of APP roles in many cases. Previous positive or negative experiences of APPs often encourage or discourage organisations or departments to consider APPs [30, 76–78]. Organisations sometimes conducted local assessments and reviews to guide planning on APP roles and continuous audits, monitoring and evaluation to continue refining the role [30, 52, 54, 77, 79–81]. *Organisational culture* such as organisational stability, power dynamics and willingness to embrace changes also influenced APP integration [30, 82, 83] and retention [70, 84]. For example, the distribution of power between medical and nursing leaders and which directorate APPs are situated within often influence their practice scope and activities [83]. In organisations that are more stable and with low staff turnover, APPs are also less likely to leave [70].

*Organisational policy and arrangement* also influenced all stages of APP role management. These included developing and updating governance, policies and protocols such as job descriptions [50, 75, 85, 86], employee management such as recruitment and onboarding processes [31, 86–89], line management structure, workspace, rota and pay arrangements [85, 90–93], opportunities for APP to engage in different specialty clinics and other opportunities [42, 94, 95] but also to ensure *clinical training resources and opportunities* are not taken away from medical doctors [89, 96, 97]. These often require a significant amount of *resources and time investment* to fully develop [98–100] and support [42, 49, 93–95, 101] APP roles and communicate transparently with the existing staff. Examples from Queensland Health in Australia indicated that the organisation spent a significant amount of time and resources consulting with medical and nursing colleges, planning workshops and presenting to all staff, producing detailed documentation and involving doctors in the interview and recruitment processes [31]. Such efforts ensured that PAs were accepted by doctors and nurses, and their benefits to the clinical team outweighed the potential concerns such as taking away opportunities for doctors and nurses themselves [31]. Articles from low- and middle-income countries often highlighted the lack of resources significantly impeded APPs’ role enactment and career progress [50, 101]. Also relevant to these areas is performance appraisals, as many organisations included this to encourage personal and professional development but often the value and impact of APP roles proved to be hard to measure and quantify [30, 50, 67, 102].

At the meso-level, the *views of other departments and teams*, as well as *APP representation within organisations*, are important. This was especially true when APP roles were proposed or developed in one specific department and physicians from other specialties actively demonstrate resistance to work with APPs, which impeded role development [37, 71] and integration [93, 103, 104]. In comparison, having APP representation across different hospital teams and practice areas as well as in organisational planning facilitated their integration [105, 106].

### Micro-level individual and interpersonal factors

*Individual APPs’ backgrounds, interests, experiences and expertise* directly influenced how they are recruited, integrated and retained. Personal interest in specific roles, specialties, work-life and family considerations often influence the supply of APPs to recruit [77, 107] and whether they would stay in the organisations [43, 94]. We also found that APPs’ prior clinical experience and practical clinical and inter-personal expertise [42, 90, 108–110] (for example NPs prior work as nurses) improved APPs’ credibility in the eyes of other healthcare team members [89, 111, 112]. Such prior work can also, however, lead to an individual’s role confusion or even conflict as they transition from previous roles into APP roles [86, 113, 114] which impedes successful integration.

*Clinical team members’ perception, understanding and relationship* with APP roles are commonly reported in the included articles as key factors across all stages of role management. This included recognition of APP competence or concern over them being ‘cheap alternatives’ that influenced the organisational decision to develop APP roles [44, 73] and processes for integration [30, 42, 75, 86, 115]. Positive or negative encounters with team members, mentors/supervisors and peers also affected integration [32, 75, 90, 116], retention and long-term career development [30, 68, 70, 117–119]. We found that relationships with clinical team members often included APPs understanding and navigating workplace professional hierarchy and power [50, 106, 117, 119, 120], and trying to negotiate roles and boundaries over time [121–124]. Another interesting factor is the *autonomy of APP roles*, as APPs often reported insufficient autonomy or others misinterpreting autonomy as meaning ‘doing everything on their own’ which negatively impacted teamwork and integration [106, 115].

Lastly, we found that *patient perceptions and preferences* often influenced organisations’ decisions to develop, recruit and integrate APPs. Interestingly, many articles reported more negative impressions of or perceived patient preferences from organisational leaders and clinical team members when developing APP roles [28, 59, 98]. In comparison, patients’ own perspectives ranged from having strong preferences for doctors, having no idea who APPs are, to being less concerned over service providers so long as they are seen [86, 122, 125, 126]. Their preferences were often related to the specific services and severity of conditions but actual encounters were commonly described more positively by patients [30, 54].

### Other emerging themes

We have also identified three emerging cross-cutting themes. First, while we focused on APP roles in general and there are similarities between different APP roles in secondary care which is doctor-centric, there were key differences between different APPs, especially PAs and NPs. How other clinical team members perceived APPs was related to the APPs’ background [73, 75, 77]. NPs usually have years of working experience as nurses before being retrained and therefore are more familiar with the healthcare settings. Other clinical team members also perceived them to be more credible which facilitated their integration [44, 77, 86, 127], whereas PAs in many countries require no prior clinical work experience and were recruited into clinical teams fresh out of training [59, 73].

Second, included articles highlighted the flexibility and fluidity of APP roles, which is both a blessing and a curse for organisations. Diverse APP roles exist within hospital settings [31, 93]; for example there were four types of clinical nurse consultants within one Australian hospital with differences in specialty, expertise and role focus [86]. Such flexibility and fluidity could ensure APP roles respond to specific local service needs, and especially for NPs the fluidity of role boundaries bridging medical and nursing roles may further improve continuity of patient care. However, this created role ambiguity and self-doubt amongst APPs [75, 106, 128, 129], confusion and conflicts with doctors and other clinical team members around APP roles and responsibilities [30, 122], and made standardisation of protocols, policies and performance appraisals challenging [30, 67, 75, 85].

Lastly, many articles emphasised that it takes significant time and resources to develop and integrate APPs roles. This included time and resources for organisation management to develop strategies and guidelines, communicate and gather input from staff, and even find a suitable APP to recruit [31, 52, 100]; for individual APPs to familiarise themselves with the clinical systems and form new identities [90, 112]; and also for other clinical team members to socialise and build trust with APPs over time [41, 42, 130].

## Discussion

Our scoping review of 273 articles summarised different factors influencing the development, recruitment, integration, retention and career development of APP roles in secondary care teams across the global literature. These spanned health system structural levels, from national and regional workforce policies and regulations, organisational and department planning, strategy and resources, to individual APP attributes and interpersonal relationships between team members.

APP roles have been developed largely to meet changing healthcare demands as well as hospitals’ increasing staff shortages [1, 5, 131]. Increasing APP supply from training programmes, work-hour restrictions on doctors and over-reliance on locum staff have prompted hospitals to consider developing and recruiting new roles to provide the much-needed continuity and stability for patients and the team [132, 133]. While the introduction of new roles has stretched traditional professional boundaries to ensure patients benefit from health care teams with better skill mixes, it has caused competition between professions, especially with junior doctors who themselves are concerned over their training opportunities, job security and gradually developing their professional identities [134–136]. Such dynamics often influence whether APP roles can be developed and successfully integrated into secondary care teams.

While more articles offered insights into the development, recruitment and integration of APP roles, only 16% investigated APP retention and career development. This could be linked to the fact that many APP roles are defined and developed catering to local needs; thus, mobility of APP in these roles is rather limited across organisations [20]. Thinking about career development is especially important for entry-level APP roles such as PA, and our review specifically highlighted the importance of organisational resources and opportunities for APPs to continuously engage in a variety of specialised clinical, training, research and quality improvement activities.

Our review has important implications for hospital managers. First and foremost, there is no one-size-fits-all scenario for APP role development, integration and retention, and organisations need to develop context-specific solutions and strategies. To ensure success, hospitals need to conduct workforce diagnoses and planning [1, 137, 138] to identify current opportunities and challenges and readiness for new roles and innovations. This can provide the foundation for establishing the value of new roles and possible career pathways envisioned for APP [139, 140]. Organisations also need to decide if, where and what APP role would be most helpful (for example PA, NP or other roles) and whether APP would take on a more generalist or specialist role [20]. Second, organisations need to recognise developing and integrating APP roles requires significant time and resource investment, often multi-year journeys. Using APP as a short-term fix without careful local planning and design can cost rather than save, fragment care, threaten quality and further worsen organisational stability, especially when services and resources are already stretched [1]. However, if carefully planned, APP could bring long-term positive changes to the clinical team including job satisfaction and interprofessional collaborations as well as improved patient outcomes [30, 110, 141]. Importantly, organisations also need clear and transparent communications of their strategies and processes. Introducing new roles could be perceived as a threat to pre-existing professional identities and jurisdictions, and further proliferation of different APP roles and titles managed by different models could further cause confusion and uncertainty for the workforce as well as for patients [131, 136]. Organisations need early and continuous engagement of local staff across departments and teams, patient representatives and external collaborators to develop and sustain APP roles.

Our findings also highlighted the importance of national policies and regulations in guiding APP role development. In countries and settings where regulation of APP roles is lacking, further clarity and guidance on professional regulation, scope of practice and career development framework is needed from national regulators in consultations with existing APP workforce, professional associations and unions, as well as patient advocacy groups [142, 143].

While this study provides a comprehensive review of APP development, recruitment, integration, retention and career development in secondary care drawing from literature across high-income and low- and middle-income countries, several limitations should be considered while interpreting these findings. Firstly, this was a scoping review and we did not, therefore, systematically assess the quality of the included articles. We also did not systematically rate our confidence in each thematic finding, but have provided the number of supporting articles for each theme and subtheme in Additional file 3. Secondly, we used an interpretative approach to data charting and collation; for example several articles had a different primary focus than workforce integration. As the review team members are primarily health systems researchers and clinicians and do not include PAs or NPs, a different research team might provide a different interpretation of the data. Lastly, we acknowledge our characterisation of APPs as a single unit could be overly simplistic. We grouped APPs because of their often overlapping scope of practice in hospital settings and it has been used by Medicare and Medicaid for coding and billing purposes, but we recognised differences between PA and NP in our analysis. In our analysis, all factors but one applied to both PA and NP roles. The exception ‘individual interest or lack of interest in advancing to APP roles’ is unique to nurses advancing to NP roles, and this factor influenced APP development and recruitment. We also acknowledge that there could be further differences within various types of advanced nursing roles such as nurse practitioners and nurse specialists [144], and we recommend future studies focus on one specific role, e.g. PA or NP, and whereas possible continue to compare and clarify the differences in these roles.

## Conclusions

In conclusion, we summarised a wide range of factors influencing the development, recruitment, integration, retention and career development of APP roles in secondary care teams. We highlighted the importance of national and regional workforce policies, organisational and departmental planning, processes and resources, as well as individual APP characteristics and interpersonal relationships with clinical team members. Organisations need to recognise that there is no one-size-fits-all scenario for developing and integrating APPs. Long-term investment with significant resource input and transparent processes is needed to develop context-specific workforce solutions and strategies to tackle evolving healthcare challenges.

### Supplementary Information

Additional file 1: Example search strategy in Ovid Embase.

Additional file 2: Characteristics of included studies.

Additional file 3: Themes, subthemes and example quotes.

Additional file 4: Summary of factors and emerging themes based on PAGER framework.

## Data Availability

All data relevant to the study are included in the article or uploaded as additional files.

## Abbreviations

APP: Advanced practice provider
NP: Nurse practitioner
PA: Physician assistant/associate
PAGER: Patterns, Advances, Gaps, Evidence for practice and Research recommendations
PRISMA: Preferred Reporting Items for Systematic reviews and Meta-Analyses
