# Supplementary material for: Factors influencing the development, recruitment, integration, retention and career development of advanced practice providers in hospital health care teams: a scoping review
Source: BMC Med. 2024 Jul 8;22:286. doi: 10.1186/s12916-024-03509-6 (PMC11232288; doi:10.1186/s12916-024-03509-6)
Supplement: Supplementary file 1 — Additional file 1: Example search strategy in Ovid Embase. [file 12916_2024_3509_MOESM1_ESM.docx]

**Additional file 1. Example search strategy in Ovid Embase**

*Search conducted April 24, 2023*

Database(s): Embase 1974 to present
Search Strategy:

| **#** | **Searches** | **Results** |
| --- | --- | --- |
| 1 | career/ | 32663 |
| 2 | exp workforce/ | 15110 |
| 3 | (career or workforce or employment or absenteeism or recruit* or retention or turnover* or leave or integrating or integration).ti,ab. | 1648011 |
| 4 | 1 or 2 or 3 | 1667421 |
| 5 | exp nurse practitioner/ | 29685 |
| 6 | ("nurse practitioner*" or "advanced practice nurs*" or "nurse clinician*" or "specialist nurs*" or "nurs* specialist*").ti,ab. | 34289 |
| 7 | ("non-physician clinician*" or "nonphysician clinician" or "clinical officer*" or "mid-level provider*").ti,ab. | 1197 |
| 8 | ("physician assistant*" or "physician associate*" or "assistant medical officer*" or "clinical assistant*" or "anaesthesia assistant*" or "anesthesia assistant*" or "anaesthesia associate*" or "anesthesia associate*" or "surgical care practitioner*").ti,ab. | 7367 |
| 9 | 5 or 6 or 7 or 8 | 53982 |
| 10 | exp secondary health care/ | 11829 |
| 11 | exp hospital/ | 1397530 |
| 12 | (hospital* or "acute care" or "secondary care" or "tertiary care" or outpatient or "clinical team*" or "emergency department*" or NHS or UK).ti,ab. | 3087612 |
| 13 | 10 or 11 or 12 | 3582534 |
| 14 | 4 and 9 and 13 | 2798 |
| 15 | limit 14 to yr="2000 - Current" | 2661 |
