# Supplementary material for: Factors influencing the development, recruitment, integration, retention and career development of advanced practice providers in hospital health care teams: a scoping review
Source: BMC Med. 2024 Jul 8;22:286. doi: 10.1186/s12916-024-03509-6 (PMC11232288; doi:10.1186/s12916-024-03509-6)
Supplement: Supplementary file 2 — Additional file 2: Characteristics of included studies. [file 12916_2024_3509_MOESM2_ESM.docx]

**Additional file 2. Characteristics of included studies**

| **ID** | **title** | **Year** | **Country** | **Cadre** | **Setting** | **Setting detail** | **Study design** | **Study design detail** | **Participants APP or others** | **Participants** | **Before or after APP onboard** | **Recruitment or development** | **Integration** | **Retention or career development** |
| --- | --- | --- | --- | --- | --- | --- | --- | --- | --- | --- | --- | --- | --- | --- |
| 6 | 'What can you do then?' Integrating new roles into healthcare teams: Regional experience with physician associates | 2019 | UK | PA | Inpatient | Five hospital sites affiliated to one trust | Mixed methods | Survey, focus group and interview | Both | Consultants, junior doctors, senior nurses, PAs | Both |  | X | X |
| 8 | A Comprehensive Onboarding and Orientation Plan for Neurocritical Care Advanced Practice Providers | 2018 | US | NP/ANP | Inpatient | One neurocritical care unit | N/A | N/A | N/A | N/A | Before |  | X |  |
| 15 | A national survey: acceptance of physician assistants and nurse practitioners in trauma centers | 2010 | US | Mixed | Emergency | National level data | Quantitative | Survey | Others | Clinical director | Both | X | X |  |
| 17 | A new kind of gatekeeper: The increasing prevalence of Advanced Practice Nurses as case managers in US hospitals | 2021 | US | NP/ANP | Mixed | National level data | Quantitative | Secondary data analysis | N/A | N/A | After |  | X |  |
| 20 | A qualitative analysis of health professionals' job descriptions for surgical service delivery in Uganda | 2014 | Uganda | CO | Mixed | National level data | Qualitative | Document review | N/A | N/A | After |  | X |  |
| 28 | A survey exploring factors affecting employment of physician associates in Ireland | 2022 | Ireland | PA | Mixed | National level data | Quantitative | Questionnaire survey | Others | Hospital management | After | X |  |  |
| 29 | A survey of nurse practitioner perceptions of integration into acute care organisations across one region in Ireland | 2022 | Ireland | NP/ANP | Inpatient | One single acute health care region | Quantitative | Survey | APP | Nurse practitioners | After | X | X |  |
| 39 | Acute Care Nurse Practitioners in an Academic Multidisciplinary ICU: Good, Bad, and Not So Ugly | 2016 | US | NP/ANP | Inpatient | One single ICU | Qualitative | Reflection | N/A | N/A | After | X | X |  |
| 40 | Acute Care Nurse Practitioners in Oncologic Critical Care: The Memorial Sloan-Kettering Cancer Center Experience | 2010 | US | NP/ANP | Inpatient | One single ICU | Qualitative | Reflection | N/A | N/A | After | X | X |  |
| 42 | Acute care nurse practitioners', physicians' and staff nurses' relationships with patients: a descriptive, comparative study | 2008 | US | NP/ANP | Mixed | One single hospital | Qualitative | Interview | Both | ACNP, patient, physician, staff nurse | After |  | X |  |
| 43 | Acute Care Pediatric Nurse Practitioners as Leaders: Perceptions, Self-Identity, and Role Congruity | 2020 | US | NP/ANP | Inpatient | One single hospital | Qualitative | Interview | APP | ACPNP | After |  | X |  |
| 49 | Advanced and specialist nursing practice: Attitudes of nurses and physicians in Israel | 2008 | Israel | NP/ANP | Mixed | 3 large hospitals and 15 community clinics | Quantitative | Survey | Others | Advanced or specialist nurse practitioner | Before | X |  |  |
| 50 | Advanced clinical practitioner role in the emergency department | 2013 | UK | Other - ACP | Emergency | One single hospital | Qualitative | Reflection | N/A | N/A | After |  | X |  |
| 53 | Advanced level nursing in England: organisational challenges and opportunities | 2015 | UK | NP/ANP | Mixed | One single hospital | Quantitative | Survey | APP | ANP | After |  | X | X |
| 56 | Advanced nurse practitioners' (emergency) perceptions of their role, positionality and professional identity | 2020 | Ireland | NP/ANP | Mixed | 10 out of 78 possible participants, no clear setting | Qualitative | Interview | APP | ANP | After |  | X |  |
| 58 | Advanced Nursing Practice and Advanced Practice Nursing roles within low and lower-middle-income countries | 2022 | Multi-country | NP/ANP | Mixed | 24 countries | Quantitative | Survey | Others | Nursing expert | N/A |  | X |  |
| 59 | Advanced nursing practice in paediatric critical care | 2015 | UK | NP/ANP | Inpatient | One single hospital | Quantitative | Survey | Others | Doctors and nurses | Before | X |  |  |
| 66 | Advanced Practice Nurses in Palliative Care: A Pivotal Role and Perspective | 2006 | US | NP/ANP | Inpatient | One single hospital | Qualitative | Reflection | N/A | N/A | After | X | X |  |
| 68 | Advanced practice nurses' organization commitment: Impact of job environment, job satisfaction, and person-organization fit | 2023 | South Korea | NP/ANP | Inpatient | One single hospital | Quantitative | Survey | APP | APN | After |  | X |  |
| 70 | Advanced Practice Nursing in Chile and the Role of the Registered Nurse: Integrating 2 Realities Through Continuous Education | 2021 | Chili | NP/ANP | Inpatient | 4 hospitals with different sizes and sectors | Qualitative | Interview | Others | Nurses | Before | X |  |  |
| 72 | Advanced practice nursing role delineation in acute and critical care: Application of the Strong Model of Advanced Practice | 2000 | US | NP/ANP | Inpatient | One single hospital with additional online recruitment | Quantitative | Survey | APP | CNS and NP | After |  | X |  |
| 80 | Advanced Practice Providers Utilization Trends in Otolaryngology From 2012 to 2017 in the Medicare Population | 2021 | US | Mixed | Mixed | National level data | Quantitative | Secondary data analysis | N/A | N/A | After |  | X |  |
| 82 | Advanced practice registered nurses and physician assistants in sleep centers and clinics: a survey of current roles and educational background | 2014 | US | Mixed | Mixed | One single hospital | Qualitative | Reflection | N/A | N/A | After | X | X |  |
| 83 | Advanced practice registered nurses' work environment perceptions in hospitals: A cross-sectional survey | 2020 | US | NP/ANP | Inpatient | All hospitals in Florida | Quantitative | Survey | APP | certified nurse practitioners (CNPs) and certified registered nurse anaesthetists (CRNAs) | After |  | X |  |
| 84 | Advanced practice role delineation within Finland: A comparative descriptive study | 2022 | Finland | NP/ANP | Mixed | 5 university hospitals | Quantitative | Survey | Both | APN, registered nurses and midwives, specialist nurses | After |  | X |  |
| 85 | Advanced providers in pediatric surgery: Evaluation of role and perceived impact | 2019 | US | Mixed | Mixed | National level data | Quantitative | Survey | Others | Paediatric surgeon | Both |  | X |  |
| 86 | Advanced registered nurse practitioners and physician assistants in the practice of pediatric neurosurgery: a clinical report | 2011 | US | Mixed | Inpatient | One single hospital | Qualitative | Reflection | N/A | N/A | After |  | X |  |
| 89 | American Association of Critical-Care Nurses' national survey of facilities and units providing critical care | 2006 | US | NP/ANP | Inpatient | National level data | Quantitative | Survey | Others | Unit managers | Both | X |  |  |
| 90 | American Association of Nurse Practitioners National Nurse Practitioner sample survey: Update on acute care nurse practitioner practice | 2018 | US | NP/ANP | Inpatient | National level data | Quantitative | Survey | APP | Acute care nurse practitioners | After |  | X | X |
| 91 | An advanced nurse practitioner service for neonates, children and young people | 2017 | US | NP/ANP | Inpatient | One single hospital | Qualitative | Reflection | N/A | N/A | After | X | X |  |
| 92 | An advanced nurse practitioner-led service-Consequences of service redesign for managers and organizational infrastructure | 2011 | UK | NP/ANP | Inpatient | One single hospital | Qualitative | Reflection | Both | Professional and non-professional | After | X | X |  |
| 93 | An analysis of job satisfaction among physician assistants in Taiwan | 2005 | Taiwan | PA | Mixed | 8 hospitals | Quantitative | Survey | APP | PA | After |  | X |  |
| 94 | An ethnographic study exploring the role of ward-based advanced nurse practitioners in an acute medical setting | 2012 | UK | NP/ANP | Inpatient | One single hospital | Qualitative | Participant observation | APP | ANP | After |  | X |  |
| 95 | An evaluation of the role of the advanced nurse practitioner on an elective orthopaedic ward from the perspective of the multidisciplinary team | 2021 | UK | NP/ANP | Inpatient | One orthopaedic unit | Qualitative | Interview | Others | Doctors and nurses | After |  | X |  |
| 97 | An exploration of the perceptions of emergency department nursing staff towards the role of a domestic abuse nurse specialist: a qualitative study | 2015 | UK | NP/ANP | Emergency | One single ED | Qualitative | Interview | Both | Nurse, specialist nurse, ED assistant | Both | X |  |  |
| 102 | Analysis of trends in nurse practitioner billing for emergency medical services: 2015-2018 | 2022 | US | Mixed | Emergency | National level data | Quantitative | Secondary data analysis | N/A | N/A | After |  | X |  |
| 108 | Assistant practitioners in palliative care: doing things differently | 2018 | UK | Mixed | Inpatient | One single hospital | Qualitative | Reflection | N/A | N/A | After | X |  |  |
| 109 | Association of British Clinical Diabetologists (ABCD): Survey of specialist diabetes care services in the UK, 2000. 2. Workforce issues, roles and responsibilities of diabetes specialist nurses | 2002 | UK | NP/ANP | Mixed | National level data | Quantitative | Survey | Others | Unit managers | After |  | X |  |
| 110 | Association of private equity ownership with increased employment of advanced practice professionals in outpatient dermatology offices | 2021 | US | NP/ANP | Outpatient | National level data | Quantitative | Survey | Others | Unit managers | Both | X |  |  |
| 112 | Attitudes of paediatric intensive care nurses to development of a nurse practitioner role for critical care transport | 2919 | UK | NP/ANP | Inpatient | One single hospital | Qualitative | Qualitative survey | Others | Nurses | Both | X | X |  |
| 113 | Barriers to Practice: Understanding Physician and Hospital Administrator Knowledge, Beliefs, and Attitudes of the Role and Scope of Practice of Acute Care Nurse Practitioners in the Acute Care Setting in Rural Montana | 2018 | US | NP/ANP | Inpatient | Two hospitals | Quantitative | Survey | Others | Doctors and hospital administrators | Before | X |  |  |
| 115 | Boundary work and the introduction of acute care nurse practitioners in healthcare teams | 2011 | Canada | NP/ANP | Mixed | Two hospitals | Qualitative | Interview, focus group and document review | Both | Inter and intraprofessional teams | After | X | X |  |
| 116 | Building a Renowned Clinical Nurse Specialist Team: Recruitment, Role Development, and Value Identification | 2019 | US | NP/ANP | Mixed | One single hospital | Qualitative | Reflection | N/A | N/A | Both | X | X |  |
| 118 | Building to make a difference: Advanced practice nurses' experience of power | 2018 | Canada | NP/ANP | Mixed | One single hospital | Qualitative | Interview | APP | APN | After |  | X |  |
| 123 | Cardiology Nurse Practitioners: Who Are We? The Development of the Role at The Mount Sinai Hospital | 2011 | US | NP/ANP | Mixed | One single hospital | Qualitative | Reflection | N/A | N/A | Both | X | X |  |
| 124 | Cardiovascular innovations: role, impact and first-year experience of a physician assistant | 2012 | UK | PA | Mixed | One single hospital | Qualitative | Reflection | N/A | N/A | After |  | X |  |
| 125 | Characteristics and perceptions of the US nurse practitioner hospitalist workforce | 2021 | US | NP/ANP | Mixed | National level data | Quantitative | Survey | APP | ANP | After |  | X |  |
| 126 | Characteristics and Role Functions of Advanced Practice Nurses in a Tertiary Pediatric Setting | 2004 | US | NP/ANP | Mixed | One single hospital | Quantitative | Survey | APP | APN | After |  | X |  |
| 129 | Clinical nurse specialist role implementation structures, processes and outcomes: Participatory action research | 2021 | Finland | NP/ANP | Mixed | One single hospital | Qualitative | Participatory action research including interviews and focus group | N/A | N/A | Both | X | x |  |
| 132 | Clinician experience, perceptions, and acceptance of paediatric complex care nurse practitioner roles | 2021 | Australia | NP/ANP | Mixed | One single hospital | Quantitative | Survey | Others | Doctors, nurses, allied health professionals | Both |  | X |  |
| 133 | Collaboration among nurse practitioners and registered nurses in outpatient oncology settings in Canada | 2012 | Canada | NP/ANP | Outpatient | One single hospital | Qualitative | Interview | Both | NP and RN | After |  | X |  |
| 136 | Communication, leadership and organizational support facilitate successful transition into practice for nurse practitioners in the emergency department | 2021 | US | NP/ANP | Emergency | One region | Quantitative | Survey | APP | NP | After |  | X |  |
| 138 | Consumer preferences regarding physiotherapy practitioners and nurse practitioners in emergency departments - a qualitative investigation | 2019 | Australia | NP/ANP | Emergency | One single hospital | Qualitative | Focus group | Others | Patient | After | X | X |  |
| 140 | Coping with a crowded ED: an expanded unique role for midlevel providers | 2002 | US | NP/ANP | Emergency | One single hospital | Qualitative | Reflection | N/A | N/A | Both | X | X |  |
| 148 | Demonstrating advanced practice provider value: Implementing a new advanced practice provider billing algorithm | 2019 | US | NP/ANP | Mixed | One single hospital | Quantitative | Secondary data analysis | N/A | N/A | N/A |  | X |  |
| 150 | Determinants of the sustained employment of physician assistants in hospitals: A qualitative study | 2016 | Netherlands | PA | Inpatient | 10 hospitals | Qualitative | Interview | Both | Doctor, resident, nurse, PA | After | X |  |  |
| 153 | Developing a neonatal workforce: role evolution and retention of advanced neonatal nurse practitioners | 2003 | UK | NP/ANP | Inpatient | APPs graduating from one course | Quantitative | Survey | APP | Advanced neonatal nurse practitioner | After |  | X | X |
| 154 | Developing an advanced nurse practitioner service in emergency care: Attitudes of nurses and doctors | 2006 | Ireland | NP/ANP | Emergency | One single hospital | Quantitative | Survey | Others | GPs, doctors and nurses | Before | X |  |  |
| 155 | Developing the nurse practitioner role in a rural Australian hospital -- a Delphi study of practice opportunities, barriers and enablers | 2009 | Australia | NP/ANP | Mixed | One single hospital | Quantitative | Delphi | Others | Doctors, nurses, patients, managers | Before | X |  |  |
| 157 | Developing the role of advanced nurse practitioners in mental health | 2010 | UK | NP/ANP | Inpatient | One single hospital | Qualitative | Reflection | N/A | N/A | Both | X |  |  |
| 161 | Development and testing of an advanced practice clinical advancement program within an academic medical center | 2021 | US | Mixed | Mixed | One single hospital | Mixed methods | Survey and focus groups | APP | NP, PA | Both | X |  |  |
| 170 | Differences and similarities in scope of practice between registered nurses and nurse specialists in emergency care: an interview study | 2020 | Norway and Finland | NP/ANP | Emergency | Two hospitals | Qualitative | Interview | Both | Nurse specialists, registered nurses and management | After |  | X |  |
| 172 | Discovering the nature of advanced nursing practice in high dependency care: a critical care nurse consultant's experience | 2004 | UK | NP/ANP | Inpatient | One single hospital | Qualitative | Reflection | N/A | N/A | After | X | X |  |
| 174 | Domains of practice and Advanced Practice Nursing in Australia | 2013 | Australia | NP/ANP | Mixed | One single hospital | Quantitative | Face-to-face survey | APP | Clinical nurse consultant | After |  | X |  |
| 175 | Domestic violence and abuse: An exploration and evaluation of a domestic abuse nurse specialist role in acute health care services | 2016 | UK | NP/ANP | Mixed | One single hospital | Qualitative | Interview | Both | Nurse and advanced practitioner nurse | After | X | X |  |
| 177 | Driven by Ambitions: The Nurse Practitioner's Role Transition in Dutch Hospital Care | 2015 | Netherlands | NP/ANP | Mixed | Several different hospitals | Qualitative | Interviews and focus groups | APP | NP | After |  | X |  |
| 179 | Dynamics and processes influencing role integration of advanced practice nurses and nurse navigators in oncology teams | 2022 | Belgium | NP/ANP | Mixed | 11 hospitals | Qualitative | Interviews and focus groups | APP | APN and oncology nurse navigators | After |  | X |  |
| 183 | Emergency nurse practitioners: the views of patients and hospital staff at a major acute trust in the UK | 2010 | UK | NP/ANP | Emergency | One single hospital | Mixed methods | Questionnaire survey and interviews | Others | Nurses, doctors, radiologist, pharmacists, patients | Before | X |  |  |
| 184 | Enacting fit in work organization and occupational structure design: The case of intermediary occupations in a dutch hospital | 2009 | Netherlands | NP/ANP | Mixed | One single hospital | Qualitative | Interview and observation | Both | Nurse, APP, doctor, admin, patients | After | X | X | X |
| 185 | Evaluating a new model of nurse-led emergency department mental health care in Australia; perspectives of key informants | 2015 | Australia | NP/ANP | Emergency | One single ED | Qualitative | Interviews and focus groups | Both | Nurses, doctors, mental health liaison nurse | Both |  | X |  |
| 191 | Evolution of Critical Care Nurse Practitioner Role Within a US Academic Medical Center | 2011 | US | NP/ANP | Inpatient | One single hospital | Qualitative | Reflection | N/A | N/A | After | X | X |  |
| 197 | Experience with physician assistants in a Canadian arthroplasty program | 2010 | Canada | PA | Inpatient | One single hospital | Quantitative | Secondary data analysis and survey | Others | Doctors, nurses and patients | After |  | X |  |
| 198 | Exploration and model development for emergency department waiting room nurse role: Synthesis of a three-phase sequential mixed methods study | 2021 | Australia | NP/ANP | Emergency | Not specifically linked to hospital | Mixed methods | Interview, observation, survey | N/A | N/A | Both | X |  |  |
| 202 | Exploring the effect of PAs on physician trainee learning: An interview study | 2019 | US | PA | Mixed | One single hospital | Qualitative | Interview | Both | PA and doctor | After |  | X |  |
| 204 | Exploring the scope of the emergency department mental health nurse practitioner role | 2007 | Australia | PA | Outpatient | One single hospital | Qualitative | Interviews and focus groups | Both | Doctor, nurse, mental health nurse practitioners | After | X |  |  |
| 206 | Factors Affecting Turnover of Advanced Practice Providers: A University Teaching Hospital Review | 2023 | US | Mixed | Inpatient | One single hospital | Quantitative | Survey | APP | PA and APRN | After |  | X | X |
| 207 | Factors associated with the job satisfaction of certified nurses and nurse specialists in cancer care in Japan: Analysis based on the Basic Plan to Promote Cancer Control Programs | 2020 | Japan | NP/ANP | Mixed | National level data | Quantitative | Survey | APP | Certified nurse specialists | After |  | X | X |
| 210 | Factors influencing the contribution of advanced practice nurses to promoting evidence-based practice among front-line nurses: Findings from a cross-sectional survey | 2011 | UK | NP/ANP | Mixed | 25 NHS trusts | Quantitative | Survey | APP | APN | After |  | X |  |
| 211 | Factors Related to Successful Transition to Practice for Acute Care Nurse Practitioners | 2016 | US | NP/ANP | Inpatient | One social media site | Quantitative | Survey | APP | ACNP | After |  | X | X |
| 212 | Factors supporting career pathway development amongst advanced practice nurses in Thailand: A cross-sectional survey | 2021 | Thailand | NP/ANP | Mixed | National level data | Quantitative | Survey | APP | APN | After |  |  | X |
| 218 | Fluid role boundaries: Exploring the contribution of the advanced nurse practitioner to multi-professional palliative care | 2015 | UK | NP/ANP | Inpatient | One hospital | Qualitative | Interview and focus group | Both | ANP, multi-professional staff, patients | After |  | X |  |
| 219 | Focus group interviews to examine the role and development of the clinical nurse specialist | 2001 | UK | NP/ANP | Mixed | 2 hospitals | Qualitative | Focus group | Others | CNS | After |  | X | X |
| 222 | From education to practice: What we can learn about the workforce from a survey of new nurse practitioners | 2019 | US | NP/ANP | Mixed | 29 NP program | Quantitative | Survey | APP | Family NP | Before | X |  |  |
| 231 | Health workforce attrition in the public sector in Kenya: a look at the reasons | 2009 | Kenya | CO | Mixed | 74 health facilities | Quantitative | Survey | Others | Facility-in-charge | After |  |  | X |
| 239 | How are acute care nurse practitioners enacting their roles in healthcare teams? A descriptive multiple-case study | 2012 | Canada | NP/ANP | Inpatient | 2 health facilities | Mixed methods | Interview, observation | Both | ACNP and other clinical and nursing team | After |  | X |  |
| 242 | How do surgical residents and non-physician practitioners play together in the sandbox? | 2005 | US | Mixed | Mixed | One program | Quantitative | Survey | Both | Surgical resident, NP and PA | After |  | X |  |
| 243 | How emergency nurse practitioners view their role within the emergency department: A qualitative study | 2016 | UK | NP/ANP | Emergency | One hospital | Qualitative | Interview | APP | Emergency nursing practitioner | After |  | X | X |
| 245 | Human resource and funding constraints for essential surgery in district hospitals in Africa: a retrospective cross-sectional survey | 2010 | 3 African countries | CO | Inpatient | 8 hospitals | Quantitative | Survey | Others | Hospital admin | After | X | X |  |
| 246 | I am quitting my job. Specialist nurses in perioperative context and their experiences of the process and reasons to quit their job | 2018 | Sweden | NP/ANP | Inpatient | 7 hospitals | Qualitative | Interview | APP | Specialist nurse | After |  |  | X |
| 247 | I Stay-Swedish Specialist Nurses in the Perioperative Context and Their Reasons to Stay at Their Workplace | 2018 | Sweden | NP/ANP | Inpatient | 4 hospitals | Qualitative | Interviews | APP | Nurse specialist (nurse anesthetist and operating room nurse) | After |  |  | X |
| 248 | Identifying practice barriers to use of adult gerontology-acute care nurse practitioners in the northern Nevada region | 2015 | US | NP/ANP | Inpatient | Subnational region | Mixed methods | Survey | Others | Managers | Before | X |  |  |
| 249 | Identity construction in nurse practitioner-patient interactions | 2015 | US | NP/ANP | Mixed | One hospital | Qualitative | Interview and conversation analysis | Both | NP and patients | After |  | X |  |
| 252 | Implementation of a medical intensive care unit acute-care nurse practitioner service | 2011 | US | NP/ANP | Inpatient | One hospital | Qualitative | Reflection | N/A | N/A | Both | X | X |  |
| 256 | Implementation of the Neonatal Nurse Practitioner Role in a Community Hospital's Labor, Delivery, and Level 1 Postpartum Unit | 2017 | Canada | NP/ANP | Inpatient | One hospital | Qualitative | Reflection and QI | N/A | N/A | Both | X | X |  |
| 257 | Implementation of the Nurse Practitioner as Most Responsible Provider model of care in a specialised mental health setting in Canada | 2022 | Canada | NP/ANP | Outpatient | One hospital | Qualitative | Reflection and QI | N/A | N/A | Both | X | X |  |
| 258 | Implementing a night-shift clinical nurse specialist | 2013 | US | NP/ANP | Inpatient | One hospital | Qualitative | Reflection | N/A | N/A | Both | X |  | X |
| 260 | Implementing the clinical nurse specialist role in a regional state psychiatric hospital | 2008 | US | NP/ANP | Inpatient | One hospital | Quantitative | Survey | APP | CNS | After |  | X |  |
| 261 | Implications of the rapid growth of the nurse practitioner workforce in the US | 2020 | US | NP/ANP | Mixed | National level data | Quantitative | Secondary data analysis | N/A | N/A | Both | X |  |  |
| 263 | Improving health care efficiency through the integration of a physician assistant into an infectious diseases consult service at a large urban community hospital | 2015 | Canada | PA | Mixed | One hospital | Quantitative | Case-control | N/A | N/A | Both | X | X |  |
| 264 | Improving patient flow in the emergency department by placing a family nurse practitioner in triage: a quality-improvement project | 2014 | US | NP/ANP | Emergency | One hospital | Qualitative | QI | N/A | N/A | Both | X | X |  |
| 270 | Integrating midlevel practitioners into a teaching service | 2006 | US | Mixed | Mixed | One hospital | Qualitative | Reflection | N/A | N/A | Both | X | X |  |
| 272 | Integrating nurse practitioners into Canadian emergency departments: a qualitative study of barriers and recommendations | 2007 | Canada | NP/ANP | Emergency | 6 Eds | Qualitative | Interview | Both | NP, doctor, RN, ED managers | After | X | X |  |
| 273 | Integrating Nurse Practitioners Into Intensive Care Units | 2016 | US | NP/ANP | Inpatient | One hospital | Qualitative | Reflection | N/A | N/A | Both | X | X |  |
| 275 | Integration and retention of American physician assistants/associates working in English hospitals: A qualitative study | 2020 | UK | PA | Mixed | Several different hospitals | Qualitative | Interview | APP | PA | After |  | X | X |
| 276 | Integration of a Physician Assistant Into an Ophthalmology Consult Service in an Academic Setting | 2018 | US | PA | Mixed | One hospital | Quantitative | Secondary data analysis and survey | N/A | N/A | Both |  | X |  |
| 282 | Integration of Palliative Care Advanced Practice Nurses Into Intensive Care Unit Teams | 2017 | US | NP/ANP | Inpatient | One hospital | Quantitative | Secondary data analysis | N/A | N/A | Both | X |  |  |
| 283 | Integration of the first physician assistants into Israeli emergency departments - The physician assistants' perspective | 2019 | Israel | PA | Inpatient | One training program | Quantitative | Survey | APP | PA | Both |  | X | X |
| 285 | Introducing a nurse practitioner into an urban Canadian emergency department | 2008 | Canada | NP/ANP | Emergency | One ED | Quantitative | Secondary data analysis | N/A | N/A | Both | X | X |  |
| 287 | Introducing physician assistants into an intensive care unit: process, problems, impact and recommendations | 2013 | UK | PA | Inpatient | One hospital | Mixed methods | Interview, survey | Both | All clinical staff | Both |  | X |  |
| 291 | Irish Respiratory Clinical Nurse Specialists' Experiences of Their Role: A Qualitative Exploration | 2018 | Ireland | NP/ANP | Mixed | 5 hospitals | Qualitative | Interview | APP | CNS | After |  | X | X |
| 293 | Is seeing a specialist nurse associated with positive experiences of care? The role and value of specialist nurses in prostate cancer care | 2008 | UK | NP/ANP | Outpatient | From 3 and 2 hospitals | Mixed methods | Interview, survey | Others | Patient | After |  | X |  |
| 297 | Job satisfaction among masters in nursing in Central and East Poland: a preliminary study | 2017 | Poland | NP/ANP | Mixed | 6 hospitals | Quantitative | Survey | APP | NP | After |  | X | X |
| 298 | Job Satisfaction and Retention of an Advanced Practice Registered Nurse Fellowship Program | 2021 | US | NP/ANP | Mixed | One hospital | Quantitative | Survey | APP | APRN | After | X |  | X |
| 300 | Labour saver or building a cohesive interprofessional team? The role of the nurse practitioner within hospitals | 2014 | Canada | NP/ANP | Inpatient | Subnational region | Qualitative | Interview and focus group | APP | NP | After |  | X | X |
| 304 | Levels of autonomy of nurse practitioners in an acute care setting | 2007 | US | NP/ANP | Inpatient | One hospital | Quantitative | Survey | APP | NP | After |  | X |  |
| 305 | the lived experience of nurse practitioner graduates' transition to hospital-based practice | 2010 | US | NP/ANP | Mixed | Subnational region | Qualitative | Interview | APP | NP | After |  | X |  |
| 308 | Making the Case for Nurse Practitioners in the Emergency Department: A Clinical Case Study | 2015 | US | NP/ANP | Emergency | One hospital | Quantitative | Secondary data analysis | N/A | N/A | After |  | X |  |
| 309 | Management of patients in the intensive care unit: Comparison via work sampling analysis of an acute care nurse practitioner and physicians in training | 2003 | US | NP/ANP | Inpatient | One hospital | Quantitative | Secondary data analysis | N/A | N/A | After |  | X |  |
| 310 | Mapping the range and scope of emergency nurse practitioner services in the Northern and Yorkshire Region: a telephone survey | 2003 | UK | NP/ANP | Emergency | 48 hospitals | Quantitative | Survey | APP | Emergency nursing practitioner | After |  | X |  |
| 311 | Mapping workforce configuration and operational models in Australian emergency departments: a national survey | 2018 | Australia | NP/ANP | Emergency | All ED | Quantitative | Survey | Others | Managers | After |  | X |  |
| 316 | Mentoring nurse practitioners in a hospital setting | 2017 | US | NP/ANP | Inpatient | One hospital | Qualitative | Interview | APP | NP | After |  | X | X |
| 319 | Mid-level providers in emergency obstetric and newborn health care: factors affecting their performance and retention within the Malawian health system | 2009 | Malawi | Mixed | Emergency | 4 hospitals | Qualitative | Interview and focus group | APP | CO and medical assistance | After |  | X | X |
| 321 | Midlevel providers in a Level I trauma service: experience at Wesley Medical Center | 2007 | US | Mixed | Inpatient | One hospital | Quantitative | Survey | Others | Patient and clinical staff | After |  | X |  |
| 323 | Modeling the Multiple Sclerosis Specialist Nurse Workforce by Determination of Optimum Caseloads in the United Kingdom | 2021 | UK | NP/ANP | Mixed | Selected hospitals | Quantitative | Survey | APP | Specialist nurse | After |  | X |  |
| 324 | Modelling the Inflammatory Bowel Disease Specialist Nurse Workforce Standards by Determination of Optimum Caseloads in the UK | 2018 | UK | NP/ANP | Mixed | National level data | Quantitative | Survey | APP | Specialist nurse | After |  | X |  |
| 325 | Motivation, money and respect: A mixed-method study of Tanzanian non-physician clinicians | 2009 | Tanzania | CO | Mixed | Subnational region | Mixed methods | Survey, interview, focus group | APP | CO and AMO | After |  |  | X |
| 326 | Multidisciplinary cancer care: Development of an infectious diseases physician assistant workforce at a comprehensive cancer center | 2010 | US | Mixed | Mixed | One hospital | Qualitative | Reflection | N/A | N/A | Both | X | X |  |
| 327 | National Study of Burnout and Career Satisfaction Among Physician Assistants in Oncology: Implications for Team-Based Care | 2018 | US | PA | Mixed | National level data | Quantitative | Survey | APP | PA | After |  | X |  |
| 328 | Negotiating jurisdiction in the workplace: A multiple-case study of nurse prescribing in hospital settings | 2014 | Netherlands | NP/ANP | Mixed | Several different hospitals | Qualitative | Interview, observation, document analysis | Both | Nurse specialist, doctors | After |  | X |  |
| 329 | Neonatal nurse practitioner role transition: The process of reattaining expert status | 2008 | US | NP/ANP | Mixed | N/A | Qualitative | Survey | APP | NNP | After |  | X |  |
| 334 | Non-physician clinicians in rural Africa: lessons from the Medical Licentiate programme in Zambia | 2017 | Zambia | CO | Mixed | N/A | Qualitative | Interview | Both | Medical licentiate, doctor, nurse, manager | After |  | X | X |
| 335 | Non-physician providers as clinical providers in cystic fibrosis: Survey of US programs | 2013 | US | Mixed | Mixed | 108 hospitals | Quantitative | Survey | Others | Program director | Both | X | X |  |
| 338 | NPs in the ICU the Vanderbilt initiative | 2012 | US | NP/ANP | Inpatient | One hospital | Qualitative | Reflection | N/A | N/A | Both | X | X |  |
| 339 | Nurse practitioner hospitalists: An empowered role | 2021 | US | NP/ANP | Inpatient | Professional society | Qualitative | Focus group | APP | NP | After |  | X |  |
| 340 | Nurse practitioner interactions in acute and long-term care: an exploration of the role of knotworking in supporting interprofessional collaboration | 2015 | UK | NP/ANP | Mixed | 6 hospitals | Qualitative | Ethnography | Both | NPs and their colleagues | After |  | X |  |
| 341 | Nurse practitioner led model of after-hours emergency care in an Australian rural urgent care Centre: health service stakeholder perceptions | 2021 | Australia | NP/ANP | Mixed | One hospital | Qualitative | Interview and focus groups | Both | NP, GP, nurse, paramedics | After | X | X |  |
| 344 | Nurse practitioner roles in pediatric emergency departments: a national survey | 2010 | US | NP/ANP | Emergency | All 205 hospitals in national association of children's hospital | Quantitative | Survey | Both | NP and charge nurse | After |  | X |  |
| 347 | Nurse practitioners and physician assistants in Dutch hospitals: their role, extent of substitution and facilitators and barriers experienced in the reallocation of tasks | 2011 | Netherlands | Mixed | Mixed | Subnational region | Mixed methods | Survey and interview | APP | PAs and NPs | After |  | X | X |
| 348 | Nurse practitioners and physician assistants in emergency medical services who billed independently, 2012-2016 | 2019 | US | Mixed | Emergency | National level data | Quantitative | Secondary data analysis | N/A | N/A | After |  | X |  |
| 350 | Nurse practitioners and physician assistants revisited: do their practice patterns differ in ambulatory care? | 2002 | US | Mixed | Ambulatory | National level data | Quantitative | Secondary data analysis | N/A | N/A | After | X | X |  |
| 355 | Nurse practitioners in medical rehabilitation settings: A description of practice roles and patterns | 2014 | US | NP/ANP | Mixed | One hospital | Qualitative | Reflection | N/A | N/A | After |  | X |  |
| 357 | Nurse practitioners in the emergency department: Establishing a successful service | 2019 | Australia | NP/ANP | Emergency | One hospital | Qualitative | Reflection | N/A | N/A | Both | X | X |  |
| 358 | Nurse practitioners leading the way: An exploratory study on the added value of nurse practitioners in outpatient care in the Netherlands | 2020 | Netherlands | NP/ANP | Outpatient | Two hospitals | Qualitative | Interview | APP | NP | After |  | X |  |
| 360 | Nurse practitioners' perceptions of their ability to enact leadership in hospital care | 2019 | Netherlands | NP/ANP | Mixed | 15 hospitals | Qualitative | Interview | APP | NP | After |  | X |  |
| 361 | Nurse practitioners' role perception, stress, satisfaction, and intent to stay at a Midwestern academic medical center | 2016 | US | NP/ANP | Mixed | One hospital | Quantitative | Survey | APP | NP | After |  | X | X |
| 362 | Nurse practitioners' work hours and overtime: How much, and under what working conditions? | 2015 | US | NP/ANP | Mixed | National level data | Quantitative | Survey | APP | NP | After |  | X |  |
| 363 | Nurse prescribing by children's nurses: Views of doctors and clinical leads in one specialist children's hospital | 2008 | UK | NP/ANP | Mixed | One hospital | Qualitative | Interview | Others | Consultants and clinical leads | After |  | X |  |
| 367 | Nurses' Perceptions of the Clinical Nurse Specialist Role Implemented in a Highly Specialized University Hospital in Spain: A Qualitative Study | 2022 | Spain | NP/ANP | Inpatient | One hospital | Qualitative | Interview | Others | Nurses | After |  | X |  |
| 371 | Nursing service innovation: A case study examining emergency nurse practitioner service sustainability | 2017 | Australia | NP/ANP | Emergency | Three hospitals | Mixed methods | Interview, survey, document review | Both | Emergency nursing practitioner, doctor, nurses, allied health staff | After |  | X |  |
| 376 | Optimal Use of Advanced Practice Providers at an Academic Medical Center: A First-Year Retrospective Review | 2023 | US | Mixed | Mixed | One hospital | Qualitative | Reflection | N/A | N/A | Both | X | X |  |
| 377 | Organizational empowerment and practice outcomes of acute care nurse practitioners in Taiwan: A national survey | 2021 | Taiwan | NP/ANP | Inpatient | National level data | Quantitative | Survey | APP | Acute care NP | After |  | X | X |
| 378 | Organizational strategies to recruit clinical nurse specialists | 2009 | US | NP/ANP | Inpatient | One hospital | Qualitative | Reflection | N/A | N/A | Both | X |  |  |
| 379 | Outcomes of adding acute care nurse practitioners to a Level I trauma service with the goal of decreased length of stay and improved physician and nursing satisfaction | 2013 | US | NP/ANP | Inpatient | One hospital | Quantitative | Secondary data analysis | N/A | N/A | Both |  | X |  |
| 383 | Paediatric nurse practitioner managed cardiology clinics: Patient satisfaction and appointment access | 2011 | US | NP/ANP | Outpatient | One hospital | Quantitative | Survey | Others | Patient | After |  | X |  |
| 384 | Parents' intentions to use paediatric nurse practitioner services in an emergency department | 2005 | Canada | NP/ANP | Emergency | One hospital | Quantitative | Survey | Others | Patient | Before |  | X |  |
| 386 | PAs in the ED: do physicians think they increase the malpractice risk? | 2011 | US | NP/ANP | Emergency | National level data | Quantitative | Survey | Others | Emergency physicians | After |  | X |  |
| 387 | PAs in the Republic of Ireland | 2023 | Ireland | PA | Mixed | National level data | Qualitative | Reflection | N/A | N/A | After | X |  |  |
| 389 | Patient and clinician experiences with an emergency department-based mental health liaison nurse service in a metropolitan setting | 2020 | Australia | NP/ANP | Emergency | One hospital | Qualitative | Interview | Others | Doctors and patients | After |  | X |  |
| 390 | Patient care, integration and collaboration of physician associates in multiprofessional teams: A mixed methods study | 2023 | UK | PA | Inpatient | One hospital | Mixed methods | Interview and survey | Both | PA, clinicians, patients | After | X | X | X |
| 391 | Patient perceptions of emergency department fast track: A prospective pilot study comparing two models of care | 2014 | Australia | NP/ANP | Emergency | One hospital | Quantitative | Survey | Others | Patients | After |  | X |  |
| 392 | Patient Perspectives on Nurse Practitioner Care in Oncology in Canada | 2017 | Canada | NP/ANP | Outpatient | One hospital | Qualitative | Interview | Others | Patients | After |  | X |  |
| 393 | Patient Preference and Perception of Care Provided by Advance Nurse Practitioners and Physicians in Outpatient Adult Congenital Clinics | 2015 | US | NP/ANP | Outpatient | One hospital | Quantitative | Survey | Others | Patients | After |  | X |  |
| 394 | Patient satisfaction with nurse practitioner care in emergency departments in Canada | 2008 | Canada | NP/ANP | Emergency | One hospital | Quantitative | Survey | Others | Patients | After |  | X |  |
| 395 | Patient Willingness to Be Seen by Physician Assistants, Nurse Practitioners, and Residents in the Emergency Department: Does the Presumption of Assent Have an Empirical Basis? | 2010 | US | Mixed | Emergency | Three hospitals | Quantitative | Survey | Both | Patients, residents and PA | Before | X |  |  |
| 399 | Patterns of utilization and evaluation of advanced practice providers on hospital medicine teams at academic medical centers | 2022 | US | Mixed | Inpatient | One hospital | Quantitative | Survey | Others | Unit leaders | After | X | X |  |
| 403 | Pediatric Nurse Practitioners: Influences on Career Choice | 2012 | US | NP/ANP | Mixed | National level data | Quantitative | Survey | APP | PNP | Both |  | X |  |
| 405 | Perceived impact on efficiency and safety of experienced American physician assistants/associates in acute hospital care in England: findings from a multi-site case organisational study | 2020 | UK | PA | Inpatient | 8 hospitals | Qualitative | Interview, document analysis | Others | Doctors, nurses, management | After | X | X |  |
| 407 | Perceptions of NPs and administrators in regard to the governing and supervision of NPs in Taiwan | 2011 | Taiwan | NP/ANP | Inpatient | One hospital | Qualitative | Interview | Both | NP, directors | After |  | X | X |
| 408 | Perceptions of nurse practitioners by emergency department doctors in Australia | 2010 | Australia | NP/ANP | Emergency | National representative | Qualitative | Interview | Others | Doctors | After | X | X |  |
| 410 | Perceptions of physicians, nurses, and respiratory therapists about the role of acute care nurse practitioners | 2004 | US | NP/ANP | Inpatient | 2 hospitals | Qualitative | Survey | Others | Doctors and nurses | After |  | X |  |
| 411 | Perceptions of role functions of psychiatric nurse specialists | 2001 | Hongkong | NP/ANP | Inpatient | 10 unites | Qualitative | Interview, observation | APP | CNS | After |  | X |  |
| 412 | Perceptions of Roles, Practice Patterns, and Professional Growth Opportunities | 2012 | US | Mixed | Mixed | One hospital | Quantitative | Survey | APP | NP and PA | After |  | X |  |
| 413 | Perceptions of the benefits and challenges of the role of advanced practice nurses in nurse-led out-of-hours care in Hong Kong: a questionnaire study | 2012 | Hongkong | NP/ANP | Mixed | National level data | Quantitative | Survey | APP | NP | Both | X |  |  |
| 414 | Perspectives and attitudes of young patients with inflammatory bowel disease: Symptoms, burden of disease and communication with their healthcare professionals | 2013 | UK | NP/ANP | Outpatient | National level data | Quantitative | Survey | Others | Patient | After |  | X |  |
| 423 | Physician Assistants and Nurse Practitioners in Rural Washington Emergency Departments | 2016 | US | Mixed | Emergency | Subnational region | Mixed methods | Survey and interview | Both | PA, NP, doctors, administrators | After | X | X |  |
| 424 | Physician assistants as physician extenders in the pediatric intensive care unit setting - A 5-year experience | 2005 | US | PA | Inpatient | One hospital | Qualitative | Reflection | N/A | N/A | After |  | X | X |
| 429 | Physician assistants in orthopedic practice: A national study | 2011 | US | PA | Mixed | National level data | Quantitative | Survey | APP | PA | After |  | X |  |
| 431 | Physician assistants working in the Department of Veterans Affairs | 2010 | US | PA | Mixed | VA hospitals | Quantitative | Secondary data analysis | N/A | N/A | After | X | X |  |
| 433 | Physician assistants: employing a new health provider in the South Australian health system | 2011 | Australia | PA | Mixed | Subnational region | Qualitative | Reflection | N/A | N/A | After | X | X |  |
| 439 | Physician associates in England's hospitals: a survey of medical directors exploring current usage and factors affecting recruitment | 2017 | UK | PA | Mixed | 71 hospitals | Quantitative | Survey | Others | Medical directors | Both | X |  |  |
| 441 | Physician associates working in secondary care teams in England: Interprofessional implications from a national survey | 2017 | UK | PA | Mixed | Professional association | Quantitative | Survey | APP | PA | After |  | X |  |
| 446 | Physician's assistants: A workforce solution for Australia? | 2011 | Australia | PA | Mixed | 4 hospitals | Mixed methods | Survey, interviews, document review | Both | PA, patients, other clinical staff | After | X | X |  |
| 447 | Physicians assistants in cardiothoracic surgery: A 30-year experience in a university center | 2005 | US | PA | Inpatient | One hospital | Mixed methods | Reflection | N/A | N/A | Both | X |  |  |
| 449 | PICU Provider Supply and Demand: A National Survey | 2018 | US | NP/ANP | Inpatient | National level data | Quantitative | Survey | Both | Senior NP and medical director | After | X |  |  |
| 450 | Pilot survey of NICU nurses' interest in the neonatal nurse practitioner role | 2005 | US | NP/ANP | Inpatient | 8 hospitals | Qualitative | Survey | Others | Nurse | Both | X |  |  |
| 453 | Practice characteristics of nurse practitioners in mental health and psychiatric settings | 2018 | US | NP/ANP | Mixed | National representative | Quantitative | Survey | APP | NP | After |  | X |  |
| 454 | Practice patterns and organizational commitment of inpatient nurse practitioners | 2015 | US | NP/ANP | Inpatient | Convenience sample of national conference | Quantitative | Survey | APP | NP | After |  | X |  |
| 465 | Pushing boundaries in paediatric intensive care: training as a paediatric retrieval nurse practitioner | 2007 | UK | NP/ANP | Inpatient | 3 participants | Qualitative | Reflection | N/A | N/A | Both | X |  |  |
| 468 | Realistic evaluation of an emergency department-based mental health nurse practitioner outpatient service in Australia | 2011 | Australia | NP/ANP | Outpatient | One hospital | Mixed methods | Realist evaluation | Both | NP. Doctor, nurse | Both |  | X |  |
| 470 | Refining the model for an emergency department-based mental health nurse practitioner outpatient service | 2008 | Australia | NP/ANP | Outpatient | One hospital | Qualitative | Group discussion | Others | Doctors and nurses | Before | X |  |  |
| 475 | Research to action: Nurse practitioners in the emergency department, Emergency Department Transition Clinic and Intravenous Therapy Clinic at Strathcona Community Hospital | 2020 | Canada | NP/ANP | Emergency | One hospital | Mixed methods | Secondary data analysis, survey, focus group discussions | Both | NP. Doctor, patients | After |  | X |  |
| 476 | Resident Work Hour Changes in Children's Hospitals: Impact on Staffing Patterns and Workforce Needs | 2015 | US | Mixed | Mixed | All 114 hospitals in national association of children's hospital | Quantitative | Survey | Others | Managers | Both | X |  |  |
| 477 | Resilience, dysfunctional behavior, and sensemaking: The experiences of emergency medicine physician assistants encountering workplace incivility | 2021 | US | PA | Emergency | Professional association | Qualitative | Interview | APP | Emergency PA | After |  | X | X |
| 480 | Rhetoric or reality? What nurse practitioners do to provide self-management support in outpatient clinics: An ethnographic study | 2016 | Netherlands | NP/ANP | Outpatient | One hospital | Qualitative | Interview, observation | Both | NP, registered nurse | After |  | X |  |
| 481 | Rheumatology nurse practitioners' perceptions of their role | 2006 | UK | NP/ANP | Mixed | Professional association | Quantitative | Survey | APP | NP, specialist nurse | After |  | X |  |
| 485 | Role of physician assistants in rural hospital settings in the Virgin Islands: A case study | 2014 | US | PA | Mixed | One hospital | Mixed methods | Observation, interviews | Both | PA, doctors, admin, patients | After | X | X |  |
| 486 | Role of the Neonatal Nurse Practitioner in the Community Hospital | 2019 | US | NP/ANP | Inpatient | National representative | Quantitative | Survey | APP | Neonatal NP | After |  | X |  |
| 487 | Role stress and job satisfaction for nurse specialists | 2007 | Taiwan | NP/ANP | Mixed | 5 hospitals | Quantitative | Survey | APP | Nurse specialist | After |  | X |  |
| 488 | Role transition: A descriptive exploratory study of assistant nurse clinicians in Singapore | 2018 | Singapore | NP/ANP | Inpatient | 6 hospitals and 2 clinics | Qualitative | Interview | APP | Assistant nurse clinicians | Before |  | X |  |
| 490 | Scope of practice and autonomy of physician assistants in rural vs. Urban emergency departments | 2014 | US | PA | Emergency | National representative | Quantitative | Survey | APP | PA | After |  | X |  |
| 493 | Self-role perception of nurse practitioners in northern Taiwan | 2011 | Taiwan | NP/ANP | Mixed | One hospital | Qualitative | Interview | APP | NP | After |  | X |  |
| 494 | Senior clinical nurse specialist pilot position in Hong Kong | 2001 | Hongkong | NP/ANP | Mixed | One hospital | Qualitative | Observation, interviews | APP | CNS | After |  | X |  |
| 496 | Shortcuts in knowledge mobilization: An ethnographic study of advanced nurse practitioner discharge decision-making in the emergency department | 2021 | UK | NP/ANP | Emergency | One hospital | Qualitative | Ethnography | Both | ANP, doctor | After |  | X |  |
| 498 | Social support and factors associated with self-efficacy among acute-care nurse practitioners | 2017 | Taiwan | NP/ANP | Inpatient | Several different hospitals | Quantitative | Survey | APP | NP | After |  | X |  |
| 500 | Specialist inflammatory bowel disease nursing in the UK: Current situation and future proofing | 2019 | UK | NP/ANP | Mixed | Professional networks | Quantitative | Survey | APP | CNS | After |  | X |  |
| 502 | State laws governing physician assistant practice in the United States and the impact on emergency medicine | 2014 | US | PA | Emergency | National level data | Quantitative | Secondary data analysis | N/A | N/A | After |  | X |  |
| 506 | Success indicators and barriers to acute nurse practitioner role implementation in four Ontario hospitals | 2001 | Canada | NP/ANP | Inpatient | Four hospitals | Quantitative | Survey | Both | ACNP, doctor, nurse, admin | After |  | X |  |
| 509 | Successful Integration of Advanced Practice Providers Into a Pediatric Academic Community Intensive Care Unit | 2020 | US | Mixed | Inpatient | One hospital | Mixed methods | Reflection | N/A | N/A | After | X | X | X |
| 510 | Supervision as a tool for building surgical capacity of district hospitals: the case of Zambia | 2020 | Zambia | CO | Mixed | 9 hospitals | Qualitative | Interview | Both | CO, doctors | After |  | X |  |
| 511 | Surgical care practitioner practice: one team's journey explored | 2011 | UK | SCP | Inpatient | One hospital | Qualitative | Reflection | N/A | N/A | Both | X | X |  |
| 515 | Survey and analysis of career planning status quo of ICU specialist nurses in Shaanxi | 2018 | China | NP/ANP | Inpatient | Subnational region | Quantitative | Survey | APP | Nurse specialist | After |  |  | X |
| 516 | Survey of patient satisfaction in a metropolitan emergency department: Comparing nurse practitioners and emergency physicians | 2009 | Australia | NP/ANP | Emergency | One hospital | Quantitative | Survey | Others | Patient | After |  | X |  |
| 517 | Symposium. Orientation program for hospital-based nurse practitioners | 2009 | US | NP/ANP | Inpatient | One hospital | Qualitative | Reflection | N/A | N/A | After | X | X |  |
| 519 | Task shifting between physicians and nurses in acute care hospitals: cross-sectional study in nine countries | 2018 | 9 countries | NP/ANP | Mixed | 9 countries | Quantitative | Survey | Both | Doctors and nurses | After | X | X |  |
| 522 | The acute care nurse practitioner role in Canada | 2010 | Canada | NP/ANP | Inpatient | National level study | Qualitative | Literature review, interviews and focus groups | Both | CNS, NP, doctor, other clinical team and policymaker | After |  | X |  |
| 523 | The Acute Care Nurse Practitioner: Challenging existing boundaries of emergency nurses in the United Kingdom | 2006 | UK | NP/ANP | Emergency | 4 hospitals | Mixed methods | Survey, interview | Others | Doctors, nurses | Before | X |  |  |
| 524 | The advanced nurse practitioner in context: Systemic processes | 2022 | Ireland | NP/ANP | Mixed | One hospital | Qualitative | Interview and focus group | Both | ANP, nurse, doctor and allied health professional | After |  | X |  |
| 528 | The application of change management principles to facilitate the introduction of nurse practitioners and physician assistants into six Ontario emergency departments | 2009 | Canada | Mixed | Emergency | 6 hospitals | Mixed methods | QI including focus group and survey | Both | PA, ACNP, NP, doctor | After |  | X |  |
| 530 | The career aspirations and expectations of student physician associates in the UK | 2019 | UK | PA | Mixed | Subnational region | Qualitative | Survey | APP | PA | Before | X |  |  |
| 532 | The changing landscape of nephrology physician assistants and nurse practitioners | 2021 | US | Mixed | Mixed | National level study | Quantitative | Secondary data analysis | APP | PA, NP | After | X | X |  |
| 533 | The clinical nurse specialist as resuscitation process manager | 2014 | US | NP/ANP | Inpatient | One hospital | Qualitative | Reflection | N/A | N/A | Both | X |  |  |
| 534 | The content of the work of clinical nurse specialists described by use of daily activity diaries | 2011 | Iceland | NP/ANP | Inpatient | One hospital | Quantitative | Structured activity diary | APP | CNS | After |  | X |  |
| 536 | The Cost of Not Training a Surgical Resident | 2021 | US | PA | Mixed | One hospital | Quantitative | Secondary data analysis | N/A | N/A | Before | X |  |  |
| 537 | The creation of a dementia nurse specialist role in an acute general hospital | 2011 | UK | NP/ANP | Inpatient | One hospital | Qualitative | Reflection | N/A | N/A | Both | X | X |  |
| 538 | The Design and Implementation of a Professional Development Program for Physician Assistants in an Academic Emergency Department | 2019 | US | PA | Emergency | One hospital | Qualitative | Reflection | N/A | N/A | After |  |  | X |
| 539 | The effects of resident work hour restrictions on physician assistant hospital utilization | 2006 | US | PA | Inpatient | National level data | Quantitative | Survey | APP | PA | After |  | X |  |
| 541 | The emerging role of the advanced practice epilepsy nurse: A comparative study between two countries | 2021 | UK and Spain | NP/ANP | Mixed | National representative | Quantitative | Survey | APP | Epilepsy nurse | After |  | X |  |
| 542 | The epilepsy specialist nurse: A mixed-methods case study on the role and activities | 2021 | Denmark | NP/ANP | Mixed | One hospital | Mixed methods | Survey, obvserbation | APP | Epilepsy specialist nurse | After |  | X | X |
| 544 | The evolution of the role of the Emergency Nurse Practitioner in Scotland: a longitudinal study | 2011 | UK | NP/ANP | Emergency | Subnational region | Quantitative | Survey | Others | Managers | After |  | X |  |
| 549 | The experience of care at nurse-led rheumatology clinics | 2012 | Sweden | NP/ANP | Outpatient | 3 hospitals | Qualitative | Interview | Others | Patient | After |  | X |  |
| 550 | The experience of working with anaesthesia associates in the United Kingdom and the impact on medical anaesthetic training | 2022 | UK | AA | Inpatient | 10 hospitals | Qualitative | Interview | APP | AA | After |  | X |  |
| 551 | The Experiences of Specialist Nurses Working Within the Uro-oncology Multidisciplinary Team in the United Kingdom | 2017 | UK | NP/ANP | Inpatient | National representative | Quantitative | Survey | APP | Specialist nurse | After |  | X |  |
| 555 | The general surgical care practitioner improves surgical outpatient streamlining and the delivery of elective surgical care | 2013 | UK | SCP | Outpatient | One hospital | Qualitative | Reflection | N/A | N/A | After | X | X |  |
| 557 | The impact of a 'resident replacement' nurse practitioner on an Academic Pediatric Neurosurgical service | 2010 | US | NP/ANP | Inpatient | One hospital | Quantitative | Secondary data analysis and survey | Others | Nurse, doctor and allied health professional | After | X | X |  |
| 558 | The impact of a structured onboarding program for newly hired nurse practitioners and physician assistants | 2023 | US | Mixed | Mixed | One hospital | Quantitative | Secondary data analysis | N/A | N/A | After |  | X | X |
| 559 | The Impact of Advanced Practice Provider Staffing on Emergency Department Care: Productivity, Flow, Safety, and Experience | 2020 | US | Mixed | Emergency | National level data | Quantitative | Secondary data analysis | N/A | N/A | After |  | X |  |
| 564 | The impact of nurse practitioners on care delivery in the emergency department: a multiple perspectives qualitative study | 2013 | Australia | NP/ANP | Emergency | Two hospitals | Qualitative | Interview | Both | NP, doctor, nurse | After |  | X |  |
| 565 | The impact of the implementation of physician assistants in inpatient care: A multicenter matched-controlled study | 2017 | Netherlands | PA | Inpatient | 34 hospitals | Quantitative | Trial | N/A | N/A | After |  | X |  |
| 569 | The involvement of physician assistants in inpatient care in hospitals in the Netherlands: a cost-effectiveness analysis | 2017 | Netherlands | PA | Inpatient | 34 hospitals | Quantitative | Economic evaluation | N/A | N/A | After | X | X |  |
| 573 | The millennial engagement, resiliency and retention study: What does your millennial advanced practice registered nurse workforce really want? | 2021 | US | NP/ANP | Mixed | One hospital | Quantitative | Survey | APP | APRN | After |  |  | X |
| 575 | The National Ambulatory Medical Care Survey: PAs and NPs in outpatient surgery | 2016 | US | Mixed | Outpatient | National level data | Quantitative | Secondary data analysis | N/A | N/A | After |  | X |  |
| 577 | The Relationship Between Practice Environment and Psychological Ownership in Advanced Practice Nurses | 2018 | US | NP/ANP | Inpatient | Subnational region | Quantitative | Survey | APP | APRN | After |  | X |  |
| 578 | The role and functions of clinical nurse consultants, an Australian advanced practice role: A descriptive exploratory cohort study | 2013 | Australia | NP/ANP | Mixed | One hospital | Mixed methods | Survey, interview | APP | Clinical nurse consultant | After |  | X |  |
| 580 | The role of advanced practice nurses in transplant center staffing | 2011 | US | NP/ANP | Mixed | Convenience sample of national conference | Quantitative | Survey | Both | APN and other clinical team members | After |  | X |  |
| 581 | The role of advanced practice providers in pediatric otolaryngology academic practices | 2013 | US | NP/ANP | Mixed | National representative | Quantitative | Survey | Others | Managers | After | X | X |  |
| 582 | The role of an advanced practice nurse (APN) in geriatric care at the emergency department | 2022 | Singapore | NP/ANP | Emergency | One hospital | Qualitative | Reflection | N/A | N/A | After |  | X |  |
| 583 | The role of nurse practitioners in hospital settings: Implications for interprofessional practice | 2011 | Canada | NP/ANP | Mixed | Subnational region | Mixed methods | Self-reporting log, focus group | APP | NP, APN | After |  | X |  |
| 586 | The role of physician assistants in a pediatric emergency department: a center review and survey | 2012 | Canada | PA | Emergency | One hospital | Mixed methods | Secondary data analysis, survey, focus group discussions | Both | Doctor, PA | After |  | X |  |
| 588 | The role of physician assistants in pediatric emergency medicine: the physician's view | 2013 | Canada | PA | Emergency | National representative | Quantitative | Survey | Others | Doctors | After |  | X |  |
| 589 | The role of physician associates in secondary care: the PA-SCER mixed-methods study | 2019 | UK | PA | Mixed | 6 hospitals | Mixed methods | Review, survey, interview, routine data | Both | PA, doctor, nurse, support staff, manager, patient | After | X | X | X |
| 595 | The role of the clinical nurse specialist | 2001 | Switzerland | NP/ANP | Mixed | One hospital | Qualitative | Reflection | N/A | N/A | After |  | X |  |
| 597 | The Role of the Clinical Nurse Specialist in Promoting Evidence-Based Practice and Effecting Positive Patient Outcomes | 2007 | US | NP/ANP | Mixed | One hospital | Qualitative | Reflection | N/A | N/A | After |  | X |  |
| 610 | The transformational journey of nurse practitioners in acute-care settings | 2010 | Canada | NP/ANP | Inpatient | 4 hospitals | Quantitative | Interview | APP | NP | After |  | X |  |
| 614 | The Use of Nurse Practitioners and Physician Assistants in Washington and Oregon Emergency Departments: A Descriptive Study of Current Practice | 2010 | US | Mixed | Emergency | Subnational region | Quantitative | Survey | Others | ED manager | Both | X | X |  |
| 616 | The value of the hospital-based nurse practitioner role: development of a team perspective framework | 2013 | Canada | NP/ANP | Mixed | 9 hospitals | Qualitative | Focus group | Both | Nurse, doctor, allied staff, manager | Before | X |  |  |
| 617 | The variance in distribution of cancer nurse specialists in England | 2011 | UK | NP/ANP | Mixed | National representative | Quantitative | Survey | Others | Managers | Both |  | X |  |
| 618 | The work setting of diabetes nursing specialists in the Netherlands: A questionnaire survey | 2008 | Netherlands | NP/ANP | Inpatient | National representative | Quantitative | Survey | APP | Diabetes nursing specialist | After |  | X |  |
| 622 | Training the future neurology workforce | 2000 | US | Mixed | Mixed | National representative | Quantitative | Survey | Others | Neurologist | Before | X | X |  |
| 626 | Trends in care by nonphysician clinicians in the United States | 2003 | US | Mixed | Outpatient | National representative | Quantitative | Secondary data analysis | N/A | N/A | After |  | X |  |
| 627 | Trends in midlevel provider utilization in emergency departments from 1997 to 2006 | 2009 | US | Mixed | Emergency | National representative | Quantitative | Secondary data analysis | N/A | N/A | After | X |  |  |
| 633 | Understanding acute care nurse practitioner communication and decision-making in healthcare teams | 2012 | Canada | NP/ANP | Inpatient | 2 hospitals | Qualitative | Observation | APP | Acute care NP | After |  | X |  |
| 634 | Understanding health professional role integration in complex adaptive systems: a multiple-case study of physician assistants in Ontario, Canada | 2020 | Canada | PA | Mixed | 13 hospitals and 6 clinics | Quantitative | Interview, document review | Both | PA, doctor, admin, other staff | After |  | X |  |
| 638 | Use of midlevel providers in US EDs, 1993 to 2005: implications for the workforce | 2010 | US | Mixed | Emergency | National representative | Quantitative | Secondary data analysis | N/A | N/A | After |  | X |  |
| 642 | Utilization and scope of practice of nurse practitioners and physician assistants in Montana | 2002 | US | Mixed | Mixed | Subnational region | Quantitative | Survey | Others | Hospital admin | After | X |  |  |
| 643 | Utilization and Workforce Integration of Physician Assistants | 2021 | US | PA | Emergency | 5 hospitals | Mixed methods | Survey and interview | Others | Managers | After | X | X | X |
| 647 | We are different: the voices of psychiatric advanced practice nurses on the performance of their roles | 2016 | Hongkong | NP/ANP | Mixed | One hospital | Qualitative | Interview | APP | APN | After |  | X | X |
| 650 | What is in a name -- patients' view of the involvement of 'care practitioners' in their operations | 2009 | UK | Mixed | Outpatient | 3 hospitals | Quantitative | Survey | Others | Patient | After |  | X |  |
| 651 | What is the contribution of physician associates in hospital care in England? A mixed methods, multiple case study | 2019 | UK | PA | Mixed | 6 hospitals | Mixed methods | Interview, observation, document review, work log | Both | PA, doctor, nurse, support staff, manager, patient | After | X | X |  |
| 652 | What Is the Geographic Distribution and Density of Orthopaedic Advanced Practice Professionals in Rural Counties? A Large-database Study | 2023 | US | Mixed | Mixed | National level data | Quantitative | Secondary data analysis | N/A | N/A | After | X |  |  |
| 654 | Why Do They Leave? Challenges to Retention of Surgical Clinical Officers in District Hospitals in Malawi | 2020 | Malawi | CO | Inpatient | Convenience sample, no specific setting | Qualitative | Interview | APP | CO | After |  |  | X |
| 656 | Work satisfaction, intent to stay, desires of nurses, and financial knowledge among bedside and advanced practice nurses | 2011 | US | NP/ANP | Inpatient | One hospital | Quantitative | Survey | APP | Clinical bedside nurse, APN | After |  |  | X |
| 659 | Working in a medicalised world: the experiences of palliative care nurse specialists and midwives | 2013 | UK | NP/ANP | Mixed | Professional registry | Qualitative | Interview | Both | CNS, midwife | After |  | X |  |
| 660 | Working with the team: strategies employed by hospital cancer nurse specialists to implement their role | 2007 | UK | NP/ANP | Mixed | 5 hospitals | Qualitative | Interview | APP | Specialist cancer nurse | After |  | X |  |
