# Supplementary material for: Factors influencing the development, recruitment, integration, retention and career development of advanced practice providers in hospital health care teams: a scoping review
Source: BMC Med. 2024 Jul 8;22:286. doi: 10.1186/s12916-024-03509-6 (PMC11232288; doi:10.1186/s12916-024-03509-6)
Supplement: Supplementary file 3 — Additional file 3: Themes, subthemes and example quotes. [file 12916_2024_3509_MOESM3_ESM.docx]

**Additional file 3. Themes, subthemes and example quotes**

*See ID for the ‘example paper’ column in additional file 2.*

**Development and recruitment**

| Level | Theme | Subtheme | Example papers | Example quote |
| --- | --- | --- | --- | --- |
| Macro (system-wide) | APP and other workforce supply (n=22) | Increasing supply of APPs but in some cases shortage of supply or lack of supply with specific training | 82, 339, 589 | *82 – US*  *“difficulties associated with finding APRNs and PAs with training in sleep medicine”* |
|  |  | Increasing supply of medical students and residents competing for positions | 184, 446 | *446 – Australia*  *“concerns did remain regarding health system capacity, given the increasing number of medical students expected to graduate in the next few years, and the corresponding increase in the number of junior doctors seeking training places. If the PA role is to be integrated in the health system in the future, it will be essential to ensure training places are available to accommodate both junior doctors and trainee PAs”* |
|  | National or regional workforce policy (n=43) | Policy and strategy guidance | 29, 589 | *29 – Ireland*  *“The Department of Health in Ireland developed a policy to support the development of a critical mass of nurse practitioner roles in targeted areas. The impact of this policy in supporting the development of nurse practitioner roles is evident in the large number of roles since 2018, particularly in older person services”* |
|  |  | Scope of practice and prescription regulations | 150, 248, 261 | *261 – US*  *“NP supply growth from 2010 to 2017 was notably slower in states with restrictive scope-of-practice regulations (100 percent) than in states with full practice authority for NPs (133 percent)”* |
|  |  | Billing and service reimbursement regulations | 248, 522 | *248 – US*  *“Half of the respondents stated that section 1848 of the Medicare reimbursement legislation has an influence on the use of AGACNPs within their facility”* |
|  |  | Workforce policy such as work hour restrictions on doctors | 112, 157, 326, 581 | *581 – US*  *“ACGME work force hours were an important inﬂuence on the utilization of NPs and PAs, with 46% of responders reporting that ACGME work force hours inﬂuenced their decision to hire a PA or an NP in the next year.”* |
|  |  | Specific funding streams for APP, including from government or charities | 108, 263, 534 | *534 – UK*  *“The Dementia Nurse Specialist (DNS) post was funded by a Strategic Workforce Investment for Tomorrow (SWIFT) grant. This supports one whole-time equivalent Band 7 RMN-qualiﬁed DNS. The post was supported by a non-recurring grant and funding was for a ﬁxed time period of 2 years. This is a common situation across the country, but it does not provide institutional stability for these innovative posts”* |
|  | Views of external stakeholders and collaborators (n=3) | Views of professional associations and hospital collaborators | 129, 150, 617 | *617 – UK*  *“There has been a lobby for more specialist nurses, usually led by cancer charities with a specific pathology focus”* |
| Meso (organisational and departmental) | Characteristics of hospitals and departments (n=13) | Specific characteristics such as academic hospitals affiliated with training institutions, urban or rural | 248, 378 | *378 – US*  *“Our successful partnering with local colleges and universities resulted in an increased number of academic CNS practices being held in the institution.”* |
|  | Organisational need and planning (n=66) | Organisational view of service gaps, for example need for service expansion, improving access | 191, 485 | *191 – US*  *“The initial recruitment of NPs was primarily triggered by the opening of a new 16-bed ICU, without any expansion of the teaching programs that provided residents for ICU service and education. The work demands had outstripped the physician labor supply. It was clear that with no additional house staff available, using allied health professionals would be necessary.”*  *485 – US*  *“administrators feeling as though the workload was not enough in every department to support a PA, a physician, or physicians”* |
|  |  | Organisational workforce shortage | 150, 264 | *150 – Netherlands*  *“a main motive for employing a PA for inpatient care was a shortage of residents because of an unfavourable geographical location or a less attractive hospital because of the nonteaching status.”* |
|  |  | Anticipated role of APP, such as flexible and less specialised role to meet patient demand, or promotion role for existing workforce, though the role might be less attractive for recruitment | 112, 627, 589 | *627 – US*  *“Simultaneously, the proportion of EDs utilizing MLPs increased from 28.3% in 1997 to 77.2% in 2006 (17% annual growth). Potential reasons for this increased MLP utilization include relatively lower wages, workforce shortages among board-certiﬁed or board-eligible physicians, and increased numbers of lower-acuity patients seeking”* |
|  |  | Anticipated value of APP, such as improving service continuity, hospital performance and quality of care and job satisfactions for others | 399, 616 | *616 – Canada*  *“There was an implicit expectation by several that the HB NP would take a leadership role in developing and enhancing hospital programs, maintaining and improving care environments, and introducing and integrating practice guidelines and evidence into patient care, although tasks related to direct patient care remained the priority”* |
|  | Local experience and evidence (n=12) | Organisational and departmental positive or negative experiences with APP | 439, 589 | *589 – UK*  *“The final positive contribution reported by two managers was that the employment of PAs in one service acted like a pilot and test bed for others to observe and consider innovative ways of addressing workforce issues or longer-term development in staffing their service”* |
|  |  | Local assessment and review | 91, 129, 589 | *91 – UK*  *“Initially, a time and motion study of workload in the out-of-hours period was conducted…* *The study was invaluable in demonstrating the aspects of care that could be assumed by experienced nursing staff. The project team was aware of growing evidence about the scope of the ANP role…* *In 2008, taking the audit findings, available evidence and strategic legislation into consideration, a full financial and non-financial* *option appraisal exercise was conducted.”*  *589 – UK*  *“Most clinicians and senior managers described decisions concerning actual posts for PAs (or others) as being departmentally driven by local assessment of need rather than within an explicit strategic framework”* |
|  | Organisational leaders’ or champions’ perception and understanding of APP role, responsibility and competence (n=24) | Perception of APP competency, working arrangement, including positive recognition of their experiences, and concerns over autonomy, working hour arrangements | 40, 112, 150, 523 | *112 – UK*  *“There was acknowledgement of an extensive knowledge base/level of experience prior to commencing training, an acceptance of professional accountability in the new role, and a recognition of the intensity of training, with an observation that RNPs were ‘possibly better prepared than the new doctors’”*  *150 – Netherland*  *“Another hindering factor that was mentioned was that PAs work only on regular base during daytime ofﬁce, and not at nightshifts or weekend shift. This has, according to the interviewees, a negative impact on the number of irregular shifts that physicians should work.”* |
|  |  | Key champions and advocates | 123, 287, 298 | *123 – US*  *“NPs were chosen to fill this gap in care (when compared with physician assistants) because of the efforts of a vice president of nursing who believed in the expanded role of the advanced practice nurse.”* |
|  | Organisational policy and arrangement (n=24) | Organisational governance, policy and protocol | 376, 446, 517 | *517 – US*  *“Before the hiring of a nurse practitioner, managers are provided with a written document that compares and contrasts role responsibilities commonly confused among several health care professionals (eg, physician assistants, medical residents, nurse coordinators). This crosswalk was developed to enhance the environment of understanding of NP practice and the role of the NP. It has helped ensure that the “right” professional was hired in the “right” position at the “right” time. ”* |
|  |  | Organisational communication and transparency | 129, 270, 446, 528 | *129 – Finland*  *“I would like to stress the importance of interdisciplinary team involvement in the success of the PAR process. All staff needs to be involved, if we would have done it any other way, we would have regressed to earlier decades”*  *446 – Australia*  *“Interview respondents acknowledged that Queensland Health had invested signiﬁcant time, care and resources into the Pilot, including consultation with medical and nursing peak bodies and colleges, full-day planning workshops, presentations to the staff at each site, the production of detailed documentation outlining the planned approach and roles, and the involvement of key medical ofﬁcers from each site in the recruitment and interview process.”* |
|  | Resources and processes for role development and recruitment (n=23) | Financial resources and time to support role development and recruitment | 183, 198, 285 | *285 – Canada*  *“resources required to select the right person and educate them to EP level of care will be significant”* |
|  |  | Recruitment process for example hiring manager and interview panel | 273, 338, 446 | *338 – US*  *“Also crucial to the program was the decision to hire a clinically experienced NP with leadership qualities to the position of assistant director. This leadership role was necessary to provide administrative oversight, which included coordination of recruitment and hiring strategies with the medical directors and NPs within their respective departments.”*  *273 – US*  *“For example, having a lead nurse practitioner helps organize the process of recruitment, interviews, orientation and integration into the unit, role development, and performance management. The leaders understand the patient care and work ﬂow responsibilities of the ICU, are familiar with the stakeholders’ vision of nurse practitioners’ role in the ICU, and represent the nurse practitioner workforce when discussions on who can do what occur.”* |
|  | Views of other departments and teams (n=5) | Support and resistance from other departments | 150, 523 | *150 – Netherlands*  *“A hindering factor which was mentioned is that it regularly happens that physicians from other medical specialities demand to consult a physician instead of a PA about a patient. Related to this, the positioning of their profession was mentioned by PAs as an inﬂuencing determinant.”* |
| Micro (individual and interpersonal) | APP individual interest and intention (n=12) | Interest or lack of interest in advancing to APP roles, **unique to nurses advancing to nurse practitioners** | 272, 450 | *450 – US*  *“The vast majority of the respondents were not interested in the NNP role…many were related to perceptions about the mismatch between responsibilities and NNP compensation. Variations in scheduling, role deﬁnitions, responsibilities, and NNP group satisfaction are found across the country and could inﬂuence a nurse’s decision to pursue the NNP role. Some nurses were not interested because they were satisﬁed with the role of NICU staff nurse.”* |
|  | Clinical team members’ perception and understanding of APP role, responsibility and competence (n=27) | Perception of APP competency and identity, including positive recognition of their ability to communicate, confusion and concern over ‘cheap alterative’ or ‘taking away training opportunities’ | 112, 287 | *112 – UK*  *“Twenty-nine per cent (6/21) portrayed mixed or negative feelings about the role, including fear, anxiety and apprehension. Concerns about team working, the creation of ‘mini doctors’ and suspicion that the impetus for role creation was driven by a shortage of PICU doctors were emphasized.”*  *287 – UK*  *“Prospective areas of difficulty identified confusion over ‘the PA role and its boundaries or lack there of’, their ‘lack of experience’ and potential ‘competition for training opportunities with junior doctors’, leading to the ‘perceived threat and even reluctance’ of staff to accept the role of PAs in the unit.”* |
|  | Patient perception or preference (n=16) | Leader and clinical team’s perceived patient preference, which is more negative | 82, 183, 485 | *183 – UK*  *“some staff members raised concerns that some patients might be misdiagnosed and wondered if the patient would always be afforded the option to see a doctor”* |
|  |  | Patient own perspectives, spanning from strong preference for doctors, no idea who they are, to less concern over provider, linked with the specific service provided | 138, 395 | *138 – Australia*  *“Participants recognized that EDs are busy, complex health-care environments that cater for people with a wide range of needs. They also recognized that EDs were under pressure to care for large numbers of people who sometimes wait long periods for care. In response, participants acknowledged that restructuring professional roles and responsibilities in ED by employing NPs and PPs was an appropriate response to changing circumstances and had the potential to improve care efficiency and quality.”*  *395 – US*  *“Patient willingness to use NPs and PAs for minor injuries or illness was more than 50%, but this changed with the moderate and major injury scenarios, with willingness ranging from 15% to 35%.”* |

**Integration**

| Level | Theme | Subtheme | Example papers | Example quote |
| --- | --- | --- | --- | --- |
| Macro (system-wide) | National or regional scope of work and service reimbursement policy (n=41) | Scope of practice and prescription regulations | 133, 275 | *275 – UK*  *“All participants impressed how not having the legal authority in England that they had in the US to prescribe medicines or order ionising radiation, had negative consequences for their role identity, with restrictions on the work they were given, and skills they were able to use. ”* |
|  |  | Billing and service reimbursement regulations | 184, 643 | *184 – Netherlands*  *“NPs had to have their decisions approved by a doctor — ‘sometimes less experienced than we are’, as NPs complained — because NP services are not reimbursed by insurance providers.”* |
|  | APP representation outside of organisations (n=6) | Representation, marketing and public image of APP role | 357, 360 | *118 – Canada*  *“At times, sharing information regarding the development, implementation, and impact of change initiatives at conferences and through publications led to changes across the country: “Other centres came here to see how we were implementing this policy and so this one thing kind of had ripple effects and it became really big in the sense that my scope of influence got big.” When the APNs’ sphere of influence extended beyond their organization in these ways, they experienced a greater sense of power, another example of “power creep.””* |
| Meso (organisational and departmental) | Organisational culture (n=6) | Power dynamic and willingness to embrace change | 92, 589, 633 | *633 – Canada*  *“The distribution of power among nursing and medical leaders and within the team and a strong hierarchical relationship affected the transfer of prescriptive authority in this study, especially if the ACNP role was aligned with the views of the dominant group. Such an alignment facilitated the transfer of medical activities to ACNPs in one case.”* |
|  | Organisational strategy and planning (n=61) | Organisational planned role and priorities for APP, for example filling rota, improving patient continuity and holistic care, or sometimes disagreement over priorities | 50, 239, 275, 334, 522, 589 | *239 – Canada*  *“The primary reasons for not enacting certain activities were the limited number of nurse practitioners available to complete the task, disagreements about role priorities between members of the leadership group, or the lack of authority to do so from the medical advisory board.”*  *50 – UK*  *“FY2 doctors change every few months and, although they may have good clinical skills, they are not experienced in the emergency department and may not be familiar with local policies and guidelines, and referral pathways. There is a strong reliance on agency staff of varying and unknown levels of skill and competence, and locum shifts are now offered to the ACP team before locums are requested.”* |
|  | Organisational policy and arrangement (n=75) | Organisational governance, policy and protocol, including job descriptions, administrative processes | 06, 179, 305, 319 | *179 – Belgium*  *“In most interviews throughout the research period, the lack of clear role definitions and therefore the existence of role ambiguity seemed and stayed one of the most important factors influencing the role integration process of ONN and APN.”*  *319 – Ireland*  *“Only 17% of those interviewed had written job descriptions. These did not always resemble actual duties undertaken and often had discretionary components, such as "...any job assigned to you." This lack of clarity, combined with chronic staff shortages and staff working across different wards and departments, means some staff members exceed their scope of practice (SOP).”*  *305 – US*  *“According to several participants, it usually took a time period of several months to*  *obtain full and unrestricted hospital credentials and privileges. According to participant #10, “I would say it took forever to get credentialed.” The slow process was attributed to hospital related internal factors as well as factors external to the hospital.”* |
|  |  | Organisational communication and transparency | 123, 129, 589 | *123 – US*  *“Communication about the role of the ACNPs is accomplished through visibility during patient rounds, involvement in interdisciplinary committee meetings, and development of poster presentations during Nurse’s Week highlighting the job description, activities, unique skills, and attributes of ACNPs. A brochure for patients was developed explaining the NP role in the management of their hospital stay.”* |
|  |  | Organisational process for onboarding and orientation | 273, 305, 329 | *329 – US*  *“The type and length of the new NNPs’ orientation was viewed as a huge factor affecting the transition process. Some felt they had a very good orientation and others felt the complete opposite making statements such as “I felt that I did not get any kind of orientation.” and “I was just thrown in.” The lack of an orientation supported comments of poor role transitions.”* |
|  |  | APP employee management such as working space, rota, workload and pay | 118, 411, 634 | *118 – Canada*  *“The location of their offices in relation to the population they served, the presence of an “open-door policy,” and being approachable influenced the APNs’ accessibility. For staff providing direct patient care, building trust was directly proportional to the degree of the APNs’ visibility in the clinical area.”*  *634 – Canada*  *“In general surgery and other inpatient settings, role clarity is more complicated due to turnover of residents, patients, surgeons/staff physicians in the midst of new consults, discharges and larger interprofessional healthcare teams.”* |
|  |  | Performance appraisals and reviews | 564, 589 | *564 – Australia*  *“Adopting a macro view in their assessment, physician ED directors questioned the measurable impact of NPs as they are such a small portion of a large total ED workforce.”* |
|  | Resources to support role activities and integration (n=22) | Resources and administrative support to enable APPs do their work and engage with others | 324, 355, 496 | *496 – UK*  *“A further barrier described by ANPs was the lack of computers available to search for clinical guidelines. There’s not enough workstations that are readily available within the department. You’re always struggling to get on a computer. ”* |
|  | Clinical training resource and opportunities (n=16) | Clinical training resources and opportunities between APPs and medical doctors | 202, 329, 550 | *550 – UK*  *“There was one example of a negative impact on medical training needs reported, where one AA had been self-allocating lists, and this had encroached upon particular modules for junior medical anaesthetic colleagues. However, this was promptly recognised and raised to the College Tutor by trainees, and resolved locally without conflict.”* |
|  | Local experience and evidence (n=19) | Organisational and departmental previous experiences with APP | 272, 577 | *272 – Canada*  *“Twenty respondents reported that clearly defining the NP role was essential to successful integration and that it was easier to integrate the NP into an ED if the staff had previous experience working with an NP.”* |
|  |  | Local ongoing audit, monitoring and evaluation | 124, 272, 357, 468 | *124 – UK*  *“The PA cardiology role was formally evaluated at the end of the one-year pilot, based on the achievements, patient case load, impact and limitations of the role, informed by an annual appraisal, in addition to monthly feedback meetings. The findings were presented to senior and junior colleagues at the trust’s medical grand round by the PA and lead consultant cardiologist and disseminated more widely to the trust chief executive and senior management team.”*  *272 – Canada*  *“Although a written job description may be helpful to set the stage for role implementation, ongoing discussions and refinement of the NP’s role were important.”* |
|  | Organisational leaders’ or champions’ perception and understanding (n=14) | Leaders’ understanding of APP role and ability to manage them | 305, 564 | *305 – US*  *“Some of the participants voiced frustration about leadership who did not clearly understand their NP role, due in part to lack of experience as an NP. Typically, a nurse administrator or nurse manager did the performance evaluation with feedback from the physicians. Several of the participants spoke negatively about administrators or managers who conducted NP performance evaluations and who had a poor understanding of the NP role and scope of practice.”* |
|  |  | Key champions and advocates | 179, 239 | *179 – Belgium*  *“ONN and APN state to search for partners who can facilitate their role integration….The ONN and APN claimed that influential partners can function as their initiator to patients, into the interprofessional team, and – mainly for APN - into the healthcare organization.”*  *239 – Canada*  *“In particular, the role of the medical and nursing leadership and the identiﬁcation of a champion for the nurse practitioner role were key factors that inﬂuenced nurse practitioner role enactment. The medical or nursing role champion that had been identiﬁed in each case helped to push that portion of the role’s development in the organization. The medical or nursing role champion promoted a common understanding of the role among team members, the medical advisory board and the Board of Directors.”* |
|  | Views of other departments and teams (n=6) | Support and resistance from other departments | 207, 496, 634 | *634 – Canada*  *“Unfortunately organizational and physician support can be undermined by other healthcare professions who may not understand the role, not accept orders written by the PA, or actively demonstrate resistance to role integration: “I know other pharmacies have a hard time understanding the role of PA and reject some prescriptions” [PA, FM], thus decreasing service delivery and efficiencies.”* |
|  | APP representation in organisations (n=8) | Having or lack of APP representation across hospital as well as in organisational planning | 116, 300 | *116 – US*  *“Clinical nurse specialists cross-shadowed in other practice areas to become familiar with the teams and practice setting…This aided in making sure that there is always adequate CNS presence within the organization, created a culture of team accountability, and ensured that staff knew they always had access to a clinical expert.”* |
| Micro (individual and interpersonal) | APP individual background and attribute (n=58) | APPs’ prior clinical experiences | 218, 283, 329 | *218 – UK*  *“From the observational and interview data, the level of personal and advanced knowledge held by ANPs was highly valued by medical staff, for example: I rely on them as part of the medical team. I rely on them not just as I would a junior doctor but I look to them for advice about other things knowing their nursing background and skills – advice about continence, mobility, pressure area care, practical matters”*  *283 – Israel*  *“This cohort of PA trainees with a paramedic background may also have benefited from an easier integration to the ED because of their familiarity with the environment from their previous work, especially working relationships with the nursing staff.”* |
|  |  | Self-confusions and conflicts between previous roles and APP roles | 177, 305, 610 | *305 – US*  *“Despite having had a good foundational nursing experience as a RN, study participants expressed a great deal of role uncertainty about themselves and their new NP roles…The transition from expert RN to novice NP evoked an array of emotions.”* |
|  | APP individual skills and expertise (n=54) | Clinical and administrative skills being (or not being) used | 170. 506 | *170 – Finland*  *“The NSs, however, indicated that their competence was not fully utilised. One NS with a specialisation in critical care noted that when working in a context that differs from the context one has trained for, it is unrealistic to expect to be able to fully use one’s competence. One NS with a specialisation in psychiatry stated that her competence was not fully utilised”*  *506 – Canada*  *“Staff nurses were asked if the ACNP role provided leadership, mentorship, a clinical resource, or was not influential for staff nurses. Seventy percent (n = 24) found the role to be most important as a clinical resource”* |
|  |  | People and managerial skills | 118, 275, 542 | *118 – Canada*  *“Teaching, mentoring, encouraging, recognizing, and acknowledging were actions identified by the APNs that lifted up others, helped others to build self-confidence and competence, and enabled them to take appropriate risk-taking behaviors. These actions improved clinical practice and broadened others’ sphere of influence.”*  *542 – Denmark*  *“ESNs act as glue for the various clinical, social, educational, and emotional dimensions. They support and improve the connection and communication among the members of the team, between the team and patients and caregivers, and between the team and external professionals (such as teachers in schools). ESNs are considered by their colleagues to foster effective collaboration.”* |
|  | Relationships and negotiations with clinical team members and peers (n=102) | Clinical team members’ perception of APP role competency | 179, 243, 275, 305, 589 | *275 – UK*  *“Contributory factors to the uncertainties included staff being naive about the PA role, poor awareness and understanding of the skills and competencies of the PAs, and confusion over how to manage their lack of authority in England to prescribe and order ionising radiation.”* |
|  |  | Professional hierarchy and power | 300, 319, 363, 408, 477 | *300 – US*  *“Yet, power influences from hospital leaders, and physicians negatively impact NP integration and full utilization. Unwarranted leader constraints of NP role enactment reduce role flexibility thus inhibiting their role evolution to match changing patient and team needs. This risks stagnation of practice potentially worsening care quality in the current environment of rapid change.”*  *408 – Australia*  *““The nursing admin/hierarchy at this hospital do not want NPs, the medical staff are quite keen on the idea”.”*  *477 – US*  *“Ernst and Rory likened their experience with incivility related to the hierarchy of medicine as being excluded from a club. “You know, that was the other thing…about being a PA in general. It it's just so, um,*  *heartbreaking that the docs just never see you in the club. You know what I mean?” (Rory, Interview 1) “I think it's kind of like an old boys club, it's like, uh, you know, physicians that have had a mutual respect for each other, that they don't have for PAs and for NPs.” (Ernst, Interview 1) ”* |
|  |  | Establishing relationships, negotiating roles and boundaries over time | 115, 390, 423, 660 | *115 – Canada*  *“The passage of time was identiﬁed by many participants. Participants described an awareness of boundary work in the team that lasted between 3 and 6 months.”*  *660 – UK*  *“To reduce ambiguity and develop a specialist nursing function, it was essential that SCNs were clear about the purpose and boundaries of their role and conveyed this to other team members, especially doctors: I’ve had to constantly re-evaluate and redeﬁne the role because it’s been so wishy-washy and there are all these other roles springing up. So we make it quite clear to the team about what we can and can’t do.”* |
|  |  | Peer relationship, role model and ‘groupness’ | 118, 179, 184, 517 | *184 – Netherlands*  *“The NPs were observed to start organizing their own schooling and to go to the canteen together”*  *517 – US*  *“Another resource available to new NPs is the Advanced Practice Nursing Executive Council. This group represents the advanced practice nurses in the institution, including NPs from each subspecialty area, clinical nurse specialists, certified nurse midwives, and certified registered nurse anesthetists. This council serves as the professional self-governance group and as a vehicle for communications within the institution’s advanced practice nursing community. New NPs are encouraged to bring issues to the attention of this group of advanced practice nursing leaders for recommendation or intervention.”* |
|  | Autonomy and relationships with supervisors (n=56) | Insufficient autonomy or too much autonomy | 243, 300 | *243 – UK*  *“The majority of participants held the opinion that nurses misunderstood the ENP role. This was clariﬁed with speciﬁc reference to misinterpreting the term autonomous: “. . .The perception is they expect us to be autonomous, as in they think autonomous means we will do everything for that patient. . .” (2, 139–140)*  *“. . .I think sometimes their perception/misunderstanding, especially among junior Nurses to what autonomous practice actually means. Autonomous practice means you should do everything, when in fact it means you make decisions and not necessarily have to do everything. . .””*  *300 – US*  *“Physician approval of the NP as an autonomous decision-maker creates acceptance of NP role legitimacy within the team, thus providing opportunity for HB NP to promote interprofessional work. When physicians do not support NP autonomy, their role is rendered subservient and unable to facilitate interprofessional collaboration and teamwork.”* |
|  |  | Supervisory and mentoring arrangement | 179, 305, 319, 485 | *305 – US*  *“In regards to learning “on the fly,” all the participants commented about the importance of having a mentor, particularly since most of them did not have a designated mentor when they started their NP role. Such a mentor could be an RN or other provider, including a physician, nurse practitioner, or physician assistant, but needed to be someone who understood NP practice, was willing to devote time to facilitating NPs’ clinical expertise, and was readily available for consultation and support.”*  *319 – Malawi*  *“Most staff members reported receiving support from senior colleagues when necessary, but supervision was reported to be extremely limited and almost exclusively negative or corrective in nature.”* |
|  | Patient perception or preference of APP (n=37) | Patients’ understanding of APP role | 305, 390 | *305 – US*  *“Patients and their families’ lack of understanding or acceptance of the NP role was described as a frustrating experience, which impacted the role transition experience for new NP, graduates”* |
|  |  | Patient experiences with APP | 357, 589 | *357 – Australia*  *“Consumer surveys demonstrated that patients were quick to recognise, accept and appreciate the new role and that medical, nursing and medical imaging staff within the ED were supportive of the role. The feedback also reﬂected that some suspicions or fears held by staff about the role had been allayed. Importantly, no negative feedback was received.”*  *589 – UK*  *“Most participants were not clear about the PA role but were very positive about the involvement of the PA and about the quality of the care they received. Not all patients or relatives knew that they had been treated by a PA, but despite this lack of understanding/awareness about the role, all patients said that they would be happy to be treated by a PA in the future.”* |

**Retention and career development**

| Level | Theme | | Subtheme | Example papers | Example quote |
| --- | --- | --- | --- | --- | --- |
| Macro (system-wide) | Career recognition, structure and pathways (n=7) | | Recognition of the role title, especially those with specialised training, by regulators or others | 153, 654 | *153 – UK*  *“A more important cause for concern relates to the feeling of some ANNPs that there is no formalised support for a deﬁned career structure, little recognition of their value as team members, and no professional recognition of the role from the nursing regulatory bodies”* |
|  |  |  | Career progression ladder and structure in the healthcare system | 319, 407, 589 | *319 – Malawi*  *“One of the most significant findings from this study is the predicament of COs, who are in the invidious position of being a cadre without a career path. They are described as crucial to the running of the health system, yet there is a widespread perception that they have been trained to a level at which they are useful, and then abandoned.”* |
| Meso (organisational and departmental) | Organisational culture (n=2) | | Organisational stability and morale | 247, 258 | *247 – US*  *“That’s why I’ve been around fora long time, because, in fact, there is quite a low turnover of staff here and it also means that the department is stable . I get my job done . everyone is healthy in the department.”* |
|  | Organisational strategy and planning (n=9) | | Organisational anticipated value and priorities for APP, for example considering them as replaceable, or prioritising clinical functions and procedures | 219, 246 | *219 – UK*  *“There seemed to be a general feeling of dissatisfaction with what they were able to achieve with the resources available and constant demands on time in not allowing all the components of the role to be allocated in equal proportions…The fact that the clinical component of their role always took priority was often reflected in the expectations and demands of the ward team.”*  *246 – Sweden*  *“The feeling of being replaceable and not being important for the workplace was also mentioned by several participants.”* |
|  | Organisational policy and arrangement (n=19) | | APP employee management such as line management, workload and pay | 06, 538, 634 | *538 – US*  *“A designated PA II director meets with individual PA IIs every 6 months to review progress and identify opportunities for expanding their project.”*  *634 – Canada*  *“In addition to dissatisfaction with their salary, cross-case analysis revealed very little employer/organizational responsiveness to consideration of incremental cost of living increases. Most PAs reported that their salaries have remained unchanged since the PA role was introduced to Ontario in 2006.”* |
|  |  |  | Performance appraisals and reviews | 219, 319 | *219 – UK*  *“Performance measurement was discussed within the groups, as a process that could facilitate personal and professional development, and thus used as a development strategy… This highlighted a number of issues for consideration such as: what to measure; what outcomes are a measurement of; from whose perspective is measurement being made, patient, family, nurse, management; what to compare CNS input with, a service at another hospital or no service at all; and how to measure qualitative interactions effectively…* *It was recognized that the CNS role is expensive and that there did need to be some way to quantify their input”* |
|  | Organisational leaders’ attitude (n=5) | | Employee relations between APP and managers | 246, 247 | *246 – Sweden*  *“One of the most crucial and decisive reasons for leaving one’s workplace was the head nurses’ attitude towards their employees. Not listening to or ignoring employees concerns, not being taken seriously, making decisions without employees involvement, not having the employees’ interest in mind in planning working schedules and being an ‘absent manager’ was described by several of the participants. This behaviour was labelled as ‘nonchalant’ or ‘dismissive’.”* |
|  | Resources and opportunities for continued employment and career development (n=26) | Financial resources to support continued employment and career development | | 634 | *634 – Canada*  *“The challenge of these variable sources is the dependency on intermittent, short stream funding and its impact on role sustainability. One Emergency Physician described the precariousness of funding PAs based on their contribution to meeting a pay-for-performance incentive to reduce wait times: “The danger is that if our [department] performance went down, then we would no longer be able to afford [our PAs] or if the province stopped the program, we would no longer be able to afford them. So our PAs live in fear every year, because they do not have stability in their jobs. They do not have contracts; they do not have job safety.” [MD, EM]”* |
|  |  | Physical resources to support work, specific to LMICs | | 325, 654 | *654 – Malawi*  *“Another issue was the lack of resources and/or infrastructure to meet demand for surgical services at the DH. This often caused postponement or cancellation of operations, leading to a backlog of patients and increased workload for the CACOs. Respondents who had left the public sector, expressed the benefits of no longer having to face these issues”* |
|  |  | Opportunities for engaging in specialty clinics, teaching and training, quality improvement, research and other activities | | 212, 275, 647 | *212 – Thailand*  *“A further important survey ﬁnding was that 87.1% of participants agreed that being highly experienced in specialist clinics was necessary for their career advancement and to the advancement of the profession (45.9% strongly agreed).”*  *275 – UK*  *“Being able to develop a speciﬁc role or specialist contribution was frequently mentioned by participants as a signiﬁcant motivator to extending their contract. Several participants described a niche role of which they were proud for having taken on responsibility within their medical/surgical team.”* |
| Micro (individual and interpersonal) | APP individual background (n=8) | | Personal characteristics such as age, family and personality | 212, 643 | *212 – Thailand*  *“93.1% of participants agreed that family inﬂuenced their career development (42.3% strongly agreed)”* |
|  | APP work experience and beliefs (n=15) | | Positive or negative experiences working as APPs such as job satisfaction, exposure and perceived impact of their role | 247, 408, 589 | *247 – Sweden*  *“The nurse specialists described experiencing excitement and stimulus in their current workplaces, even after working at the same workplace for many years. No one day was the same as the next, meaning that one could still learn new things every day. There was a stimulating variety in one’s work tasks and that brought pleasure to one’s work.”*  *408 – Australia*  *“We have had positive experience with nurse practitioners… they have completed the training and then decided not to pursue it…they felt the repetitive nature of the work was too restrictive”.”* |
|  | Relationship with clinical team members and peers (n=11) | | Positive or negative experiences with mentors, colleagues and peers | 246, 247, 424, 477 | *424 – US*  *“One reason offered for departure at exit interviews has been friction generated by the resident-PA interface and by the nurse-PA interface. This friction arises from confusion over professional roles and may be speciﬁc to our institution.”* |
