# Supplementary material for: Factors influencing the development, recruitment, integration, retention and career development of advanced practice providers in hospital health care teams: a scoping review
Source: BMC Med. 2024 Jul 8;22:286. doi: 10.1186/s12916-024-03509-6 (PMC11232288; doi:10.1186/s12916-024-03509-6)
Supplement: Supplementary file 4 — Additional file 4: Summary of factors and emerging themes based on PAGER framework. [file 12916_2024_3509_MOESM4_ESM.docx]

**Additional file 4. Summary of factors and emerging themes based on PAGER framework**

| Pattern | Advances | Gaps | Evidence for practice | Research recommendations |
| --- | --- | --- | --- | --- |
| Macro level factors influencing development and recruitment | Growing understanding of changes of workforce supply and workforce policy on APP role development and recruitment | There are few studies in relation to general public’s perception | Clear policy guidance on professional regulation, scope of practice, and service reimbursement contribute to APP role development and recruitment | Research is needed to explore external stakeholder, collaborator and general public view on APP and how this influenced role development and recruitment |
| Macro level factors influencing integration | Increasing number of studies showing how changes in scope of practice and billing regulations improved integration | Similarly few studies in relation to general public’s perception | Clear policy guidance on professional regulation, scope of practice, and service reimbursement contribute to APP role integration | Research is needed to explore external stakeholder, collaborator and general public view on APP and how this influenced role integration |
| Macro level factors influencing retention and career development | Some studies especially from low- and middle-income countries showing the importance of role recognition and career progression ladder | There is a paucity of research at this level | Evidence to emerge from future research | Research is needed to address this significant gap in the literature regarding system-level factors influencing APP retention and career development |
| Meso level factors influencing development and recruitment | Relatively large number of studies examining the role of organisational and departmental factors | Relatively few studies provided rich context and process of APP role development and recruitment | The importance of context-specific solutions and strategies, significant time and resource input, and need for clear and transparent communications | Greater attention needs to be given to elaborate the contexts and processes of APP role development and recruitment |
| Meso level factors influencing integration | Large number of studies examining the role of organisational and departmental factors, especially in relation to organisational strategy, planning, policy and arrangement | Similarly, relatively few studies provided rich context and process of APP role integration | Similarly, the importance of context-specific solutions and strategies, significant time and resource input, and need for clear and transparent communications | Greater attention needs to be given to elaborate the contexts and processes of APP role integration |
| Meso level factors influencing retention and career development | Relatively small number of studies pointing to the need for organisational strategy and sufficient resources for retention and career development | Limited number of studies and no factor specific to APP retention and career development, as compared with general workforce retention | Evidence to emerge from future research | Research is needed to address this significant gap in the literature regarding organisational-level factors influencing APP retention and career development |
| Micro level factors influencing development and recruitment | Growing number of studies pointing to importance of views from clinical team members and patients | More studies referring to patient perception and preference are hospital managers and clinical team members’ perception of such | Organisations need clear and transparent processes to take clinical team members’ perceptions into consideration | Research is needed to understand and compare clinical team members’ perception of patient preference and actual patient preference |
| Micro level factors influencing integration | Relatively large number of studies analysing the relationship between APPs and their supervisors, clinical team members | Relatively few studies provided rich context and process for role negotiation and eventual integration outcome | Integrating APPs into clinical team would require time for APPs to establish relationships, negotiate roles and boundaries over time | Greater attention needs to be given to elaborate the contexts, processes and outcomes of APP role integration |
| Micro level factors influencing retention and career development | Relatively small number of studies pointing to how interpersonal relationship influence retention and career development | Limited number of studies and no factor specific to APP retention and career development, as compared with general workforce retention | Evidence to emerge from future research | Research is needed to address this significant gap in the literature regarding individual and interpersonal-level factors influencing APP retention and career development |
| Other – key difference between APP roles | Several studies comparing PA and NP roles pointing out the difference in their background and training influence their own experiences and how others perceive them | Very limited number of studies examining APP roles aside from PA and NPs | NP and PA roles are different and organisations should develop and recruit specific APP positions based on need and context | Future research should focus on PA or NP role separately. Research is needed to understand other APP roles such as anaesthesia associates, surgical care practitioners, and also to compare between different advanced nursing roles |
| Other – flexibility and fluidity of APP roles | Some studies highlighting the diverse APP roles within hospitals and their benefits and challenges | Relatively few studies provided rich context and process of such role development and integration | Diverse APP roles exist within hospital settings to based on local need but could cause confusions | Greater attention needs to be given to elaborate the contexts, processes and outcomes of APP role development and integration |
| Other – time and resources required | Relatively large number of studies highlighting the time and resource required for hospital management, individual APPs and clinical team members to develop and integrate APP into teams | Relatively few studies provided rich context and process | Organisations need to recognise developing and integrating APP roles requires significant time and resource investment, often multi-year journeys | Greater attention needs to be given to elaborate the contexts, processes of APP role development and integration |
